# Long-term effects of early-life rumen microbiota modulation on dairy cow production performance and methane emissions

**DOI:** 10.3389/fmicb.2022.983823

**Published:** 2022-11-08

**Authors:** Hanna Huuki, Miika Tapio, Päivi Mäntysaari, Enyew Negussie, Seppo Ahvenjärvi, Johanna Vilkki, Aila Vanhatalo, Ilma Tapio

**Affiliations:** ^1^Department of Agricultural Sciences, University of Helsinki, Helsinki, Finland; ^2^Production Systems, Genomics and Breeding, Natural Resources Institute Finland (Luke), Jokioinen, Finland; ^3^Production Systems, Animal Nutrition, Natural Resources Institute Finland (Luke), Jokioinen, Finland

**Keywords:** rumen function, microbiome modulation, microbiome establishment, heifer, dairy cow

## Abstract

Rumen microbiota modulation during the pre-weaning period has been suggested as means to affect animal performance later in life. In this follow-up study, we examined the post-weaning rumen microbiota development differences in monozygotic twin-heifers that were inoculated (T-group) or not inoculated (C-group) (*n* = 4 each) with fresh adult rumen liquid during their pre-weaning period. We also assessed the treatment effect on production parameters and methane emissions of cows during their 1^st^ lactation period. The rumen microbiota was determined by the 16S rRNA gene, 18S rRNA gene, and ITS1 amplicon sequencing. Animal weight gain and rumen fermentation parameters were monitored from 2 to 12 months of age. The weight gain was not affected by treatment, but butyrate proportion was higher in T-group in month 3 (*p* = 0.04). Apart from archaea (*p* = 0.084), the richness of bacteria (*p* < 0.0001) and ciliate protozoa increased until month 7 (*p* = 0.004) and anaerobic fungi until month 11 (*p* = 0.005). The microbiota structure, measured as Bray–Curtis distances, continued to develop until months 3, 6, 7, and 10, in archaea, ciliate protozoa, bacteria, and anaerobic fungi, respectively (for all: *p* = 0.001). Treatment or age × treatment interaction had a significant (*p* < 0.05) effect on 18 bacterial, 2 archaeal, and 6 ciliate protozoan taxonomic groups, with differences occurring mostly before month 4 in bacteria, and month 3 in archaea and ciliate protozoa. Treatment stimulated earlier maturation of prokaryote community in T-group before month 4 and earlier maturation of ciliate protozoa at month 2 (Random Forest: 0.75 month for bacteria and 1.5 month for protozoa). No treatment effect on the maturity of anaerobic fungi was observed. The milk production and quality, feed efficiency, and methane emissions were monitored during cow’s 1^st^ lactation. The T-group had lower variation in energy-corrected milk yield (*p* < 0.001), tended to differ in pattern of residual energy intake over time (*p* = 0.069), and had numerically lower somatic cell count throughout their 1^st^ lactation period (*p* = 0.081), but no differences between the groups in methane emissions (g/d, g/kg DMI, or g/kg milk) were observed. Our results demonstrated that the orally administered microbial inoculant induced transient changes in early rumen microbiome maturation. In addition, the treatment may influence the later production performance, although the mechanisms that mediate these effects need to be further explored.

## Introduction

Global demand for food is increasing, but reduction of greenhouse gas emissions from the agricultural sectors is required to mitigate climate change (FAO, 2019). One way the livestock sector can contribute to the reduction of greenhouse gas emissions is by increasing animal’s efficiency in production. Ruminants are adapted to utilize plant material that is indigestible to many monogastric animals, including humans, due to symbiotic interaction with the microbiota inhabiting the rumen. The efficiency of digestion is linked to both animal genetics (Brito et al., 2020) and characteristics of microbial ecosystem in the gut (Kruger Ben Shabat et al., 2016). The function of microbiota can be improved by, e.g., optimizing the feeding or adding feed supplements (Beauchemin et al., 2020; Erickson and Kalscheur, 2020), by modulating the microbial communities with antimicrobials or pro-and prebiotics (Cameron and McAllister, 2016; Bąkowski and Kiczorowska, 2021). However, the fully established gut microbiota is resistant to changes, and often reverts to pre-manipulation state shortly after the end of the treatment (Weimer, 2015; Weimer et al., 2017). On the contrary, modulation of rumen microbiota while it is still developing in young ruminants may present a window of opportunity to affect animal performance or health later in life (Yáñez-Ruiz et al., 2015; Malmuthuge and Guan, 2017; Huws et al., 2018; Meale et al., 2021).

Many early-life rumen modulation studies have reported short-to medium-term effects of the treatments on the microbiota development and/or phenotypic characteristics although only a few studies have followed animals long after the treatment ended. Natural versus artificial rearing management practices were found to improve the microbial establishment in young ruminants (Abecia et al., 2014a, 2017; Belanche et al., 2019a,b). Belanche et al. (2019b) showed that natural nurturing of lambs with ewes accelerated the rumen microbiota development and eased the transition to solid diet. These lambs also retained higher fungal diversity that was suggested to contribute to better feed digestibility and growth. The pre-weaning diet influence on the rumen microbiota establishment was demonstrated by Dill-McFarland et al. (2019). They showed that feeding starter, silage, or mixed feed to calves during the pre-weaning period induced differences in bacterial, archaeal, and anaerobic fungal communities up to 2 years of age, but no later differences in milk production efficiency or overall efficiency of the animals were observed. On the contrary, Cristobal-Carballo et al. (2021) found no long-lasting effect of pre-weaning diet on microbial community structure later in life. The direct inoculation of live or processed microbial fluids as means of rumen modulation demonstrated promotion of microbial community establishment during and after weaning, with positive effect on weaning health and growth (e.g., Cersosimo et al., 2019; Belanche et al., 2020; Palma-Hidalgo et al., 2021; Park et al., 2021; Rosa et al., 2021). Park et al. (2021) demonstrated that the inoculation induced differences in community composition persisted at least 3 weeks after the end of the treatment. Studies that aimed to reduce methane emissions through antimicrobial and other feed supplements have been successful in showing medium (Abecia et al., 2013, 2014b; de Barbieri et al., 2015a,b; Lyons et al., 2017) to long-term (Meale et al., 2021) treatment effects. The observations by Meale et al. (2021) that calves treated with 3-NOP early in life had long-term reduced methane emissions suggest that rumen microbiome pre-programming is possible.

For our study, we recruited monozygotic twin calves to eliminate possible differences in response to the treatment caused by the host genetics. During the pre-weaning period, we repeatedly treated half of them with fresh rumen liquid inoculation from a feed efficient adult cow. We demonstrated that the inoculation enhanced the maturation of bacterial, archaeal, and ciliate protozoan communities, but not of rumen anaerobic fungi. Moreover, the treatment had a positive impact on animal growth, and it tended to increase the feed intake during the pre-weaning period (Huuki et al., 2022). The aim of this follow-up study was to examine the impact of early life rumen modulation on the rumen microbiota development post-weaning until animals reached 12 months of age. The second aim was to assess the possible long-term effects on animal production performance and methane emissions during their first lactation. We hypothesized that the treatment would continue to influence the microbial community development after the treatment ended and would lead to improved production efficiency later in life.

## Materials and methods

### Animals and experimental design

Four pairs of identical twin female calves produced by embryo splitting were separated into individual pens (147 cm × 172 cm) after birth and were randomly assigned to either a treatment (T-group) or a control (C-group) group as described by Huuki et al. (2022). Starting from the 2^nd^ week, the T-group calves received an oral dose of fresh rumen liquid, obtained from a fistulated adult cow, ranked previously as feed efficient animal (Mäntysaari et al., 2012). During weeks 2 and 3, the oral dose was 5 ml, given 3 times a week. During weeks 4–8, the dose was increased to 10 ml. The inoculations were stopped after weaning. The microbial composition of the inoculum and the effect of inoculum on calves’ microbiota establishment during the pre-weaning period have been reported by Huuki et al. (2022). The calves were weaned at the age of 8 weeks and the animals from both the T- and C-groups were placed into a group pen and housed together first at the Natural Resources Institute Finland (Luke) research barn, and later in Viking Genetics breeding facility (Hollola, Finland) until their first calving. Right after weaning the calves were offered silage and concentrate (Pikkumullin herkku, Raisio agro, Finland) supplement *ad libitum*. After moving to breeding facility, the weight gain of the heifers was controlled to avoid fattening. The goal for growth was to reach 280 kg by 10 ± 0.5 months of age. The weight gain was maintained at maximum of 750 g/d until 18 months old, and maximum of 550 g/d after 18 months of age by offering 2 different forage mixes with different metabolizable energy levels (~9 or ~ 10 MJ/kg DM). The feed was offered once a day. Heifers remained in good general health throughout the experiment. Weight was estimated monthly by measuring hearth-girth with a weight tape measurer (H. Hauptner und Richard Herberholz GmbH & Co. LP, Germany). The monthly weight gain was calculated by subtracting the previous month’s weight from the current weight. Two months before calving the heifers returned to Luke’s research barn. The cows were fed grass silage and concentrate mix, provided through the feeding stations. After calving the proportion of concentrate in the diet was adjusted based on the stage of lactation and the digestibility of the grass silage. When the concentration of digestible organic matter in dry matter (DM) of silage was between 680 and 700 g/kg DM, the concentrates were offered so that the proportion of concentrate in the diet DM became 52% during lactation days 1–150, 47% during days 151–250 and 37% thereafter. The amount of concentrate decreased or increased 2%-units for each 10 g /kg DM increase or decrease in digestibility of grass silage. Adjustment of cow’s individual concentrate offering was based on measured daily silage DM intake of the cow. On average the proportion of concentrate in the diet of twin cows was 49.2%. The composition of feeds is presented in Supplementary Table S1. During the first lactation month, two cows from the C-group and one from the T-group were treated with Penovet vet (Boehringer Ingelheim Animal Health Nordics A/S, Denmark) and Carepen vet (aniMedica GmbH, Germany) because of clinical signs of mastitis.

### Rumen sample collection

Rumen samples from heifers were collected monthly starting from month 2 until animals reached 12 months of age. During the 1^st^ lactation, rumen samples were collected from each cow once when they were 147 ± 11 (Average ± SD) days in milk. Rumen fluid from heifers was collected between 1,000 and 1,100 h before feeding *via* the esophageal PVC tube (10/16 or 12/18 mm inner/outer diameter) and from cows using Ruminator (Profs Products, Wittybreut, Germany). Sample preparation for VFA analysis was performed as described by Ahvenjärvi and Huhtanen (2018). In brief, the rumen liquid was filtered through two layers of cheesecloth, and a 5-mL aliquot was mixed with 0.5 ml of saturated mercuric (II) chloride solution and 2 ml of 1 M sodium hydroxide solution and stored at −20°C for later VFA analysis with gas chromatography (Huhtanen et al., 1998). For microbial analysis, 0.5 ml rumen liquid aliquots were snap-frozen in dry ice and stored at −80°C until DNA extraction.

### DNA extraction, sequencing

The total DNA was extracted from 0.5 ml of rumen liquid with a method combining bead-beating and column extraction as described by Rius et al. (2012). Universal primers 515F and 806R (Caporaso et al., 2011) targeting the V4 region of the 16S rRNA gene were used for bacterial and archaeal amplicon sequencing. Primers 316F and 539R targeting the 18S rRNA gene area were used for ciliate protozoa (Sylvester et al., 2004), and primers Neo 18SF and Neo5.8SR targeting the ITS1 region (Edwards et al., 2008) were used for amplicon sequencing of anaerobic fungi. The sequencing libraries were prepared as described by Huuki et al. (2022) and sequenced in Finnish Functional Genomics Centre (Turku, Finland) on Illumina MiSeq platform by using 2 × 250 bp chemistry for bacteria and archaea, and 2 × 300 bp for ciliate protozoa and anaerobic fungi.

### Sequence data processing

The demultiplexing of sequences, adapter removal, and sorting sequences by barcode were performed by the sequencing center. Bacterial and archaeal sequencing data were processed using QIIME 2 (Bolyen et al., 2019). Briefly, quality control, filtering of chimeric reads, and clustering of bacterial sequences into amplicon sequence variants (ASV) were performed using DADA2 (Callahan et al., 2016). The ASVs with less than 2 reads in total were removed. The bacterial ASV taxonomy was assigned using the SILVA 138 database (Quast et al., 2013) and the archaeal taxonomy was assigned using the RIM-DB database (Seedorf et al., 2014). The ciliate protozoa and anaerobic fungi sequence data were processed using QIIME v. 1.9.1 (Caporaso et al., 2010) as described by Huuki et al. (2022). In short, the reads were joined using SeqPrep and filtered for quality (>Q20) and length (protozoa: 220–300 bp; anaerobic fungi: 350–410 bp). Open reference OTU clustering (97% similarity) was performed with UCLUST, and chimeric reads were removed with usearch61 (Edgar, 2010). The OTUs with less than two reads were removed. The taxonomy was assigned with BLAST using ciliate protozoa reference database (Kittelmann et al., 2015), and anaerobic fungi ITS reference database v. 3.5, released on 9^th^ of December 2019 (Koetschan et al., 2014). The sequence reads are available in the NCBI Sequence Read Archive under BioProject PRJNA713003 with corresponding BioSample Accessions SAMN28869958 - SAMN28870037.

### Quantitation of microbial communities

The quantities of bacteria, archaea, ciliate protozoa, and anaerobic fungi were estimated with qPCR by quantifying rRNA gene copy numbers of each taxonomic group in 1 ng of extracted DNA. The quantitation of archaea, ciliate protozoa, and anaerobic fungi was performed as described by Huuki et al. (2022). The bacteria were quantified by amplifying the 16S rRNA gene area (279 bp) using primers UniF (3′ - GTG STG CAY GGY TGT CGT CA - 5′) and UniR (3′ - ACG TCR TCC MCA CCT TCC TC - 5′; Maeda et al., 2003). Amplification reaction (10 μl) contained 1 x Power SYBR Green PCR master mix (Applied Biosystems by Thermo Fischer Scientific, Life Technologies Ltd., United Kingdom), 0.2 μM of each primer, and 10 ng of DNA. The amplification was done in a Viia7 thermocycler (Applied Biosystems), with denaturation at 95°C for 10 min, 40 cycles of denaturation at 95°C for 15 s, annealing at 60°C for 30 s, and extension at 72°C for 30 s. A melt curve analysis was performed after amplification with denaturation at 95°C for 15 s, annealing at 60°C for 1 min, and denaturation at 95°C for 15 s, with a ramp increment of 0.4°C. A more detailed description of methods is provided in Supplementary material 1.

### First lactation production data

Twin cows calved between August 2019 and January 2020, when cows were 23–29 months old. The body weight (BW, kg) of the cows was recorded daily after each milking using an automatic scale. Daily milk yield (kg/day) and dry matter intake (kg/day) were monitored throughout the lactation period. Morning and evening milk samples for analyses of fat, protein, and lactose were taken once on the lactation weeks 2 and 3 and every fourth week thereafter. Milk analyses were provided by Valio Ltd. milk laboratory in Seinäjoki using MilkoScan FT+ spectrometer (Foss Electric, Hillerød, DK). Daily average milk fat, protein, and lactose content were calculated based on the morning and evening milk yields. Energy-corrected milk yield (ECM) was calculated as proposed by Sjaunja et al. (1990). Metabolizable energy intake (MEI, MJ/d) and average residual energy intake (REI, ME MJ/d) were calculated for each lactation month, except when cows were on summer pasture. REI was calculated by fitting a multiple linear regression model with ECM, metabolic BW (BW^0.75^), and piecewise regressions of BW gain or BW loss on the MEI. Thereafter, the daily residuals from the prediction equations were defined to be REI (Mäntysaari et al., 2012). Cows with low REI values are defined as efficient.

### Methane emissions

The daily methane (CH_4_) production was measured for the first 5 months after calving by photoacoustic IR spectroscopy (PAS) with F10 multigas analyzer (Gasera Ltd., Turku, Finland) as previously described by Negussie et al. (2017). Briefly, the gas analyzer was attached to two feeding stations and was recording the CH_4_ and CO_2_ gas contents of cow’s breath while the animal was visiting the feeding station. The average daily CH_4_ production was calculated for each lactation month from measurements of daily visits from both stations. Two T-group cows and one C-group cow were missing the data from lactation month 1, and one T-group cow from month 2. For one T-group cow, only data from lactation months 1 and 2 were available.

### Statistical analyses

To explore treatment and age effect on the changes in microbial community structure, between-sample diversity was evaluated as Bray-Curtis dissimilarities following Hellinger transformation and visualized using principal coordinate analysis (PCoA) with *Phyloseq* (McMurdie and Holmes, 2013) R package. The significance for the effect of age, treatment, and treatment × age interaction was estimated using distance-based permutational multivariate analysis (Adonis) as implemented in *vegan* R package (Oksanen et al., 2019) and the pairwise comparisons between age × treatment with Permutational multivariate analysis of variance with False discovery rate adjustment with *RVAideMemoire* R package (Herve, 2020). The effect of host genetics on microbial community composition was estimated from the Bray-Curtis distance matrix. Significant differences between months were estimated by comparing the average distances between twins to distances between not related heifers with Kruskal–Wallis test, and pairwise comparison with Wilcoxon signed-rank test.

The taxonomic differences between the groups were evaluated at genus level for bacteria and at species level for the rest. The significance of age and treatment × age interaction was evaluated using ANOVA. Apart from the archaea dataset, the taxa with less than 0.1% relative abundance in the dataset were filtered out. All data were log(1 + x) transformed for normalization before the analysis.

Microbial community alpha diversity in T- and C-groups was estimated using Shannon and Simpson indices, and the richness as number of observed OTUs/ASVs as implemented in *Phyloseq.* The data were evenly subsampled to 9,600 reads for bacteria, 54,394 reads for ciliate protozoa, 25,369 reads for anaerobic fungi, and 115 reads for archaea. The statistical analysis was performed with Linear Mixed Model GLIMMIX in SAS 9.4 (SAS Institute Inc., Cary, NC, United States). Treatment, age, and the treatment × age interaction were treated as fixed effects. Pair was treated as random while age (month) as a repeated effect. Based on fit statistics, the compound symmetry covariance structure was chosen for alpha diversity measures. The difference in variance between groups was considered in bacteria and archaea alpha diversity models. The Simpson index was (x)^20^ transformed for anaerobic fungi and log(x) transformed for archaea, to achieve the normal distribution of residuals, and least square means were later reverse transformed. The effects were estimated using Residual Maximum Likelihood (REML) method and were declared significant at *p* ≤ 0.05. The alpha diversity estimates during the lactation period were compared between the groups using Wilcoxon signed-rank test.

Microbial quantities, measured as gene amplicon copy numbers, were tested using Linear Mixed Model as described above. Based on the Model Fit Statistics, the covariance structure was set to compound symmetry for archaea and ciliate protozoa, and to spatial power for bacteria and anaerobic fungi to improve the model fit.

The rumen VFA proportions (mmol/1,000 mol) until 12 months of age, production performance data (BW, weight gain, dry matter intake (DMI), REI, ECM, methane emissions and milk fat, protein, and lactose proportions) were analyzed using Mixed procedure in SAS v 9.4 with fixed and random effects as above. Pair and interaction of pair and age were treated as random effects with production performance data. Spatial power covariance structure was selected for VFA proportions and autoregressive covariance structure for production data. Milk somatic cell counts (SCC) were log(x) transformed to meet model assumptions and analyzed with the program GLIMMIX using Linear Mixed Model approach. The unequal variance of residuals between groups was considered. The total VFA concentration and individual VFA proportions from the first lactation period were compared between the groups using Wilcoxon signed-rank test.

Random Forests regression was used to regress rarefied ASVs at 9,700 sequences per sample against their chronological age using default parameters as implemented in R package *randomForest* with ntree – 10,000 (Liaw and Wiener, 2002). The statistically significant OTUs were identified by Boruta algorithm in R package *Boruta* (Kursa and Rudnicki, 2010). Analysis was done with default confidence level (*p* = 0.01) but increasing the ‘*maxRuns’* to 1,000. Of the tentatively significant OTUs, those with higher median Z-score than the best shadow OTU were included as implemented in ‘*TentativeRoughFix’*-function. The C-group heifers were used in training the model to estimate the microbial maturity of the animals. A thin plate regression spline (Wood, 2003) was fit between microbiota age and chronological age of the animal separately for C-and T-groups as implemented in R-package *mgcv* (Wood, 2017). To analyze the contribution of specific OTUs on the model prediction, the approximate Shapley values were calculated with *fastshap* (Greenwell, 2021).

## Results

After quality filtering and removal of rare sequences, there were a total of 1,796,922 bacterial, 29,461 archaeal, 7,296,045 ciliate protozoan, and 6,156,516 anaerobic fungal sequences from the pre-parturition period, with an average number of 22,462 ± 6,067, 368 ± 152, 92,355 ± 13,382, and 81,007 ± 17,188 reads per sample, respectively. The data of bacteria, archaea, ciliate protozoa, and anaerobic fungi from lactation period contained a total of 258,120, 5,199, 447,438, 515,997 reads with an average of 32,265 ± 10,020, 650 ± 204, 55,930 ± 38,364, 64,500 ± 24,997 reads per sample, respectively.

### Treatment had minor effect on the archaea community

**Figure 1:**
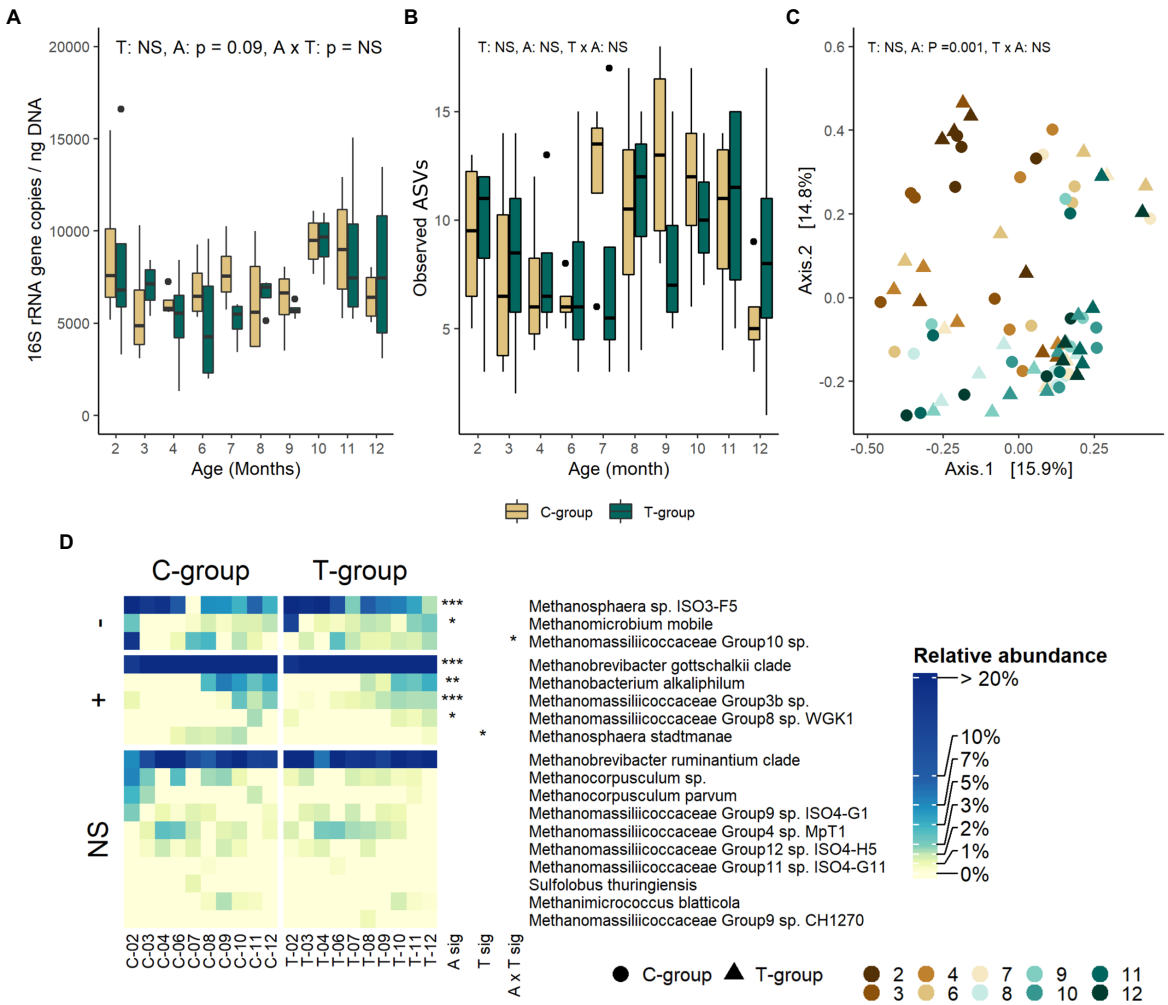
The rumen archaeal community in treatment (T-group) and control (C-group) heifers at 2–12 months of age. **(A)** The rumen archaea 16S rRNA gene copy numbers, **(B)** the number of observed ASVs, **(C)** principal coordinate analysis visualization of Bray-Curtis community distances, and **(D)** the relative abundances of rumen archaea species. Only taxa with statistical significances are shown. The taxa are grouped by increasing (+) or decreasing (−) trend with time, or no significant effects (NS). Statistical significance of Treatment (T-sig), Age (A-sig), and Treatment × Age interaction (T × A sig): *p* < 0.05: *, *p* < 0.01: **, *p* < 0.001: ***.

Treatment or interaction of age and treatment had no effect on archaeal 16S rRNA gene copy numbers (Figure 1A), ASV richness (Figure 1B), alpha diversity indexes (Supplementary Figure S1), or community distances (Figure 1C). At a taxonomical level, treatment affected the relative abundance of two species (Figure 1D; Supplementary Table S2). C-group had a higher overall abundance of *Methanosphaera stadtmanae* (treatment: *p* = 0.018) and a higher abundance of *Methanomassiliicoccaceae* group 10 sp. in month 2 (interaction: *p* = 0.04). After month 3, differences between the groups were no longer observed.

### Archaea community develops with age

The rumen archaeal quantity, estimated as 16S rRNA gene copy numbers, tended to decrease until month 9, and then increased again (Age: *p* = 0.084; Figure 1A). The archaeal ASV richness numerically decreased after month 2 and increased again after month 6, reaching the highest values in the C-group in month 7 and in the T-group in month 8 (Figure 1B). Similarly, Shannon and Simpson diversity indexes numerically decreased between months 2–6 and increased after month 6 reaching the highest values in month 7 in the C-group and month 8 in the T-group (Supplementary Figure S1). Age explained 20% of the variance in Bray–Curtis distances (treatment: *R*^2^ = 0.012, *p* = 0.400; Age: *R*^2^ = 0.2, *p* = 0.001; Interaction: *R*^2^ = 0.105, *p* = 0.403; Figure 1C). The community at month 2 was distant from the later months, but between months 3 and 10 the variation in the community composition was high and no clear separation into age groups was observed. Among the 18 archaeal species detected, *Methanosphaera* sp. ISO3-F5, *Methanobrevibacter gottschalkii* clade, and *Mbb. ruminantium* clade (71–93%) were the most abundant in both groups (Supplementary Table S2; Figure 1D). *Methanosphaera* sp. ISO3-F5 and *Methanomicrobium mobile* had their highest abundances in month 2 in both groups, but the abundance reduced by month 3. *Mbb*. *gottschalkii* became the predominant species from month 3 onward, while *Methanobacterium alkaliphilum*, *Mmc*. Group 3b sp. and *Mmc*. Group8 sp. WGK1 did not become abundant before heifers reached 8 months of age.

### Treatment effect on bacterial community gradually diminished

**Figure 2:**
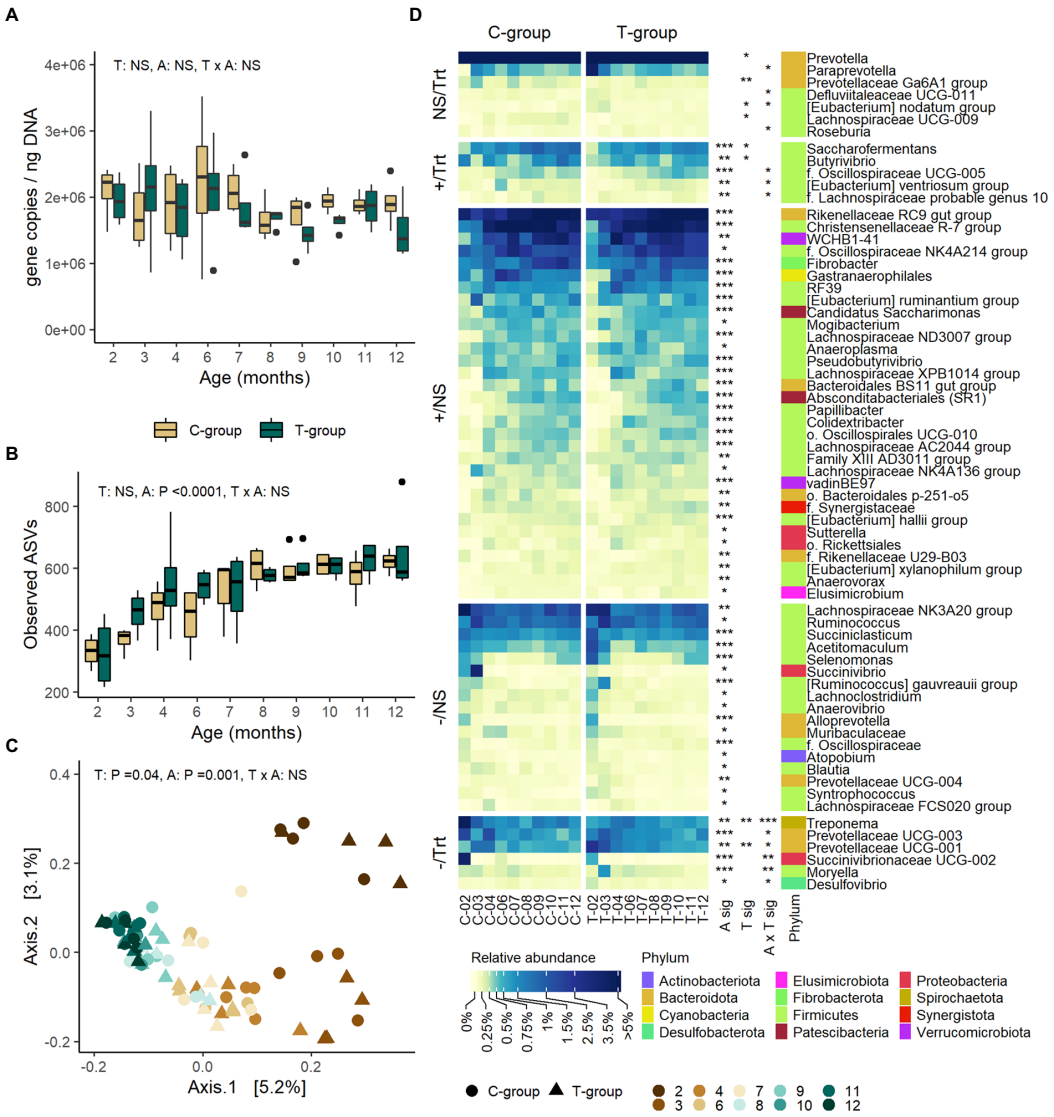
The rumen bacterial community in treatment (T-group) and control (C-group) heifers at 2–12 months of age. **(A)** The rumen bacteria 16S rRNA gene copy numbers, **(B)** the number of observed ASVs, **(C)** principal coordinate analysis visualization of Bray-Curtis community distances, and **(D)** the relative abundances of rumen bacterial genera with abundance >0.1%. Only taxa with statistical significances are shown. The taxa are grouped by increasing (+) or decreasing (−) trend with time, and with significant treatment effects (Trt) or no treatment effects (NS). Statistical significance of Treatment (T-sig), Age (A-sig) and Treatment × Age interaction (T × A sig): *p* < 0.05: *, *p* < 0.01: **, *p* < 0.001: ***.

Treatment or interaction of age and treatment had no significant effect on the rumen bacteria 16S rRNA gene copy numbers measured with RT-qPCR (Figure 2A), the ASV richness (Figure 2B), or alpha diversity metrics (Supplementary Figure S1). Treatment induced minor differences in bacterial community composition, but the effect of treatment only explained 1.4% of the variance (Adonis; treatment: *R*^2^: 0.014, *p* = 0.04) (Figure 2C). Overall, the T-group had higher abundances of *Prevotella* (treatment: *p* = 0.033), *Prevotellaceae* Ga6A1 group spp. (*p* = 0.008), *Prevotellaceae* UCG-001 spp. (*p* = 0.005), (*Eubacterium*) *nodatum* group (*p* = 0.023), and *Lachnospiraceae* UCG-009 spp., while *Butyrivibrio* (*p* = 0.019) and *Saccharofermentans* (*p* = 0.011) were less abundant as compared to C-group (Figure 2D; Supplementary Table S4). Most of the differences between the groups were detected during months 2 and 3, after which the communities became more alike (Figure 2D; Supplementary Table S4). In month 2, the T-group had higher abundances of *Prevotellaceae* spp. (*p* < 0.001), *Paraprevotella* (Tukey: *p* = 0.004), and *Desulfovibrio* (*p* = 0.005), while lower abundances of genus *Treponema* (Tukey *p* < 0.0001) and an unidentified genus affiliated with *Succinivibrionaceae* (*p* < 0.001), as compared to C-group. After month 2, only minor differences wereobserved in the taxonomic composition between the groups, which were mainly related to genera within Firmicutes phylum.

### Bacteria community develops with age

The rumen bacterial 16S rRNA gene copy numbers remained at a similar level from month 2 to month 12 (Age: *p* = 0.727; Figure 2A). ASV richness increased until month 7 in both groups (*p* < 0.0001) and then stabilized (Figure 2B). Shannon diversity increased until month 7 (Age: *p* < 0.0001), while Simpson’s evenness index was low in month 2, but no longer increased after month 3 (Age: *p* < 0.0001; Supplementary Figure S1). As indicated by Adonis analysis of Bray–Curtis dissimilarities, age explained 16.5% of the variation in the bacterial community structure (treatment: *R*^2^ = 0.014, *p* = 0.04; age: *R*^2^ = 0.165; *p* = 0.001; interaction: *R*^2^ = 0.106, *p* = 0.04; Figure 2C). The first axis separated the communities by age in months 3–6, but not from month 7 onward. The second axis separated the month 2 from the rest. The age differences between the communities were due to decreasing relative abundances of phyla Bacteroidota, Proteobacteria, and Spirochaetota and increasing abundances of Firmicutes, Fibrobacterota, Patescibacteria, and Verrucomicrobiota (Anova age: *p < 0.05*; Supplementary Table S3). At the genus level, 60 of the 94 observed genera (> 0.1% abundance) were affected by age, of which 23 decreased and 37 increased in abundance over time (Figure 2D; Supplementary Table S4). More than half of the taxa affected by age (36 genera) were affiliated with Firmicutes. The differences in bacterial community structure between months 2 and 3 were related to reducing abundances of *Lachnospiraceae* NK3A20 group, *Succiniclasticum*, *Acetitomaculum*, *Selenomonas*, *Lachnoclostridium*, *Alloprevotella*, *Succinivibrio*, and *Succinivibrionaceae* UCG-002, while *Lachnospiraceae* NK4A136 group, (*Eubacterium*) *ruminantium* group and RF39 became more abundant in month 3. Between months 4 to 9 the changes in taxonomic composition were mainly caused by increasing abundance of WCHB1-41, *Christensenellaceae* R-7 group, *Saccharofermentas*, *Rikenellaceae* RC9 gut group, and *Fibrobacter*. *Rikenellaceae RC9* gut group, *Absconditabacteriales* (SR1) sp., *Papillibacter,* and *Pseudobutyrivibrio* were among the last genera to stabilize in abundance after month 7.

To assess early life modulation effect on the rumen prokaryote community maturation during the post-weaning period, we applied the Random Forest analysis. The Random Forest prediction of rumen microbiome age, based on the 29 bacterial and archaeal ASVs, revealed that the treatment accelerated early post-weaning microbiome succession. In month 4, the maturity of rumen bacteria in the T-group was by 0.75 months ahead of the maturity of C-group (Supplementary Figure S2A). The main gradual maturation-related changes caused by the treatment were assessed by calculating the approximate Shapley values for prediction on month 4. They showed that the low abundance ASVs affiliated with, e.g., *Methanobrevibacter millerae* and some *Prevotella* ASV*s*, and a high abundance of other *Prevotella* ASVs strongly reduced the predicted age, while a higher abundance of, e.g., *Pseudobutyrivibrio*, other *Prevotella*, *Methanobrevibacter*, and *Clostridiales bacterium* strongly increased the predicted age of T-group (Supplementary Figure S2B). To summarize the patterns on ASVs identified to have an impact on age estimates, several *Christensenellaceae R-7* group ASVs were most abundant between 4–6 months of age, and some *Prevotella* and *Lachnospiraceae* NK3A20 group ASVs increased in abundance with age, while other *Prevotella* and *Succinivibrionaceae* UCG-002 ASVs reduced in abundance with age (Supplementary Figure S2C).

### Treatment effect on ciliate protozoa community diminished

**Figure 3:**
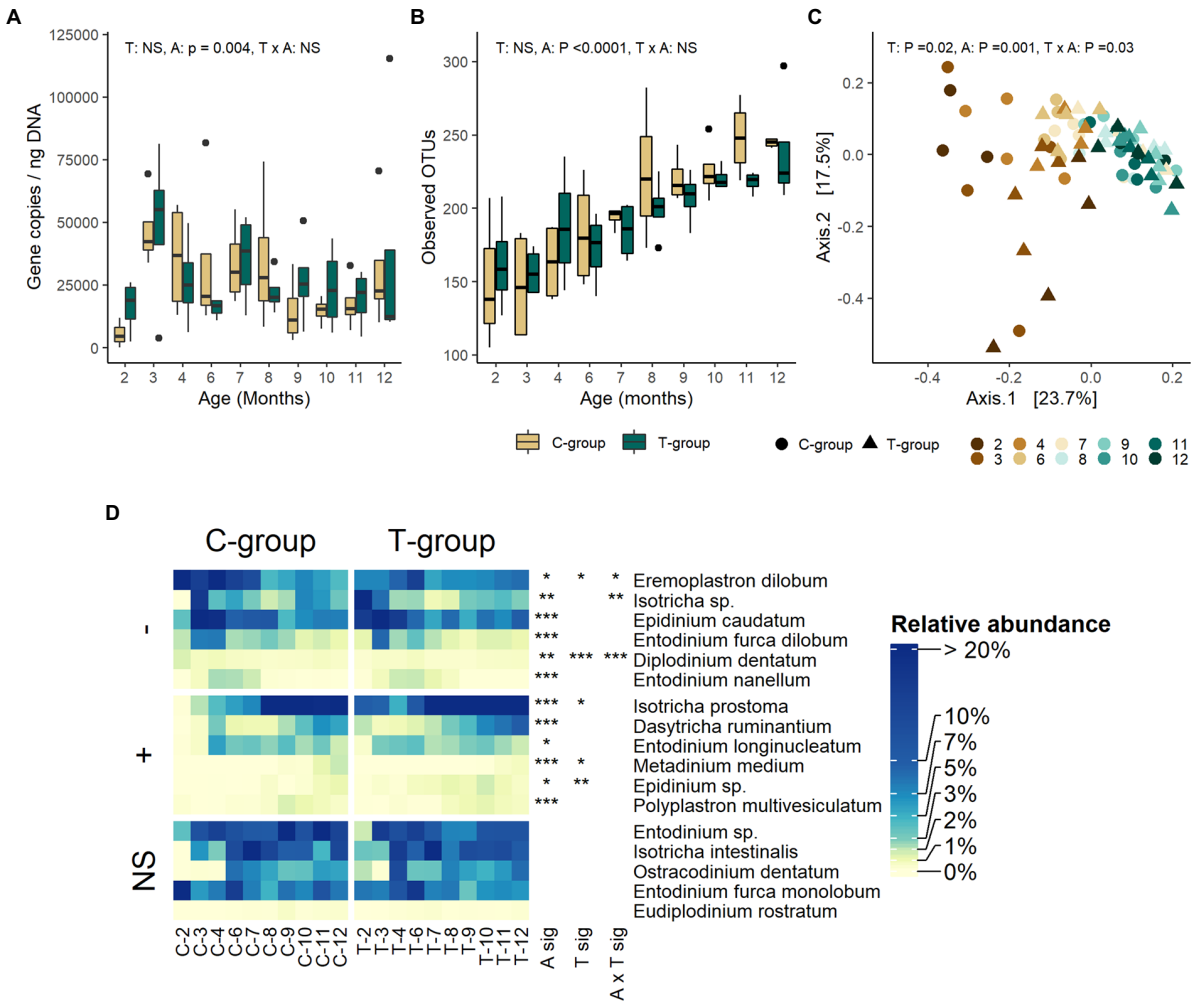
The rumen ciliate protozoa community in treatment (T-group) and control (C-group) heifers at 2–12 months of age. **(A)** The rumen protozoa 18S rRNA gene copy numbers, **(B)** the number of observed OTUs, **(C)** Principal coordinate analysis visualization of Bray-Curtis community distances, and **(D)** the relative abundances of protozoa species. Only taxa with statistical significances are shown. The taxa are grouped by increasing (+) or decreasing (−) trend with time, and no significant effects (NS). Statistical significance of Treatment (T-sig), Age (A-sig), and Treatment × Age interaction (T × A sig): *p* < 0.05: *, *p* < 0.01: **, *p* < 0.001: ***.

The ciliate protozoa copy numbers (Figure 3A) or the number of observed OTUs (Figure 3B) were not significantly affected by the treatment. However, the effect of treatment was visible in the Bray-Curtis distances, which separated the groups until month 3, but not anymore thereafter (treatment: *R*^2^ = 0.0229, *p* = 0.016; interaction: *R*^2^ = 0.1114, *p* = 0.031) (Figure 3C). The management of calves in individual pens during the pre-weaning period resulted in differences in the ciliate protozoa community composition between the groups. All but one C-group heifer received ciliate protozoa by month 2, having a community with 6 abundant (>1%) species, but were highly dominated by *Eremoplastron dilobum* and *Entodinium furca monolobum.* On the contrary, the T-group was richer, inhabited by 10 species and dominated by *Isotricha* sp.*, Epidinium caudatum,* and *Isotricha prostoma* (Supplementary Table S5; Figure 3D). The Shannon and Simpson indexes were significantly lower in the C-group in month 2 (interaction: *p* < 0.05), but in month 3 when the heifers were moved to a group pen, the differences between the groups disappeared (Supplementary Figure S1).

### Ciliate protozoa community develops with age

The ciliate protozoan 18S rRNA gene copy numbers were low in month 2 but dramatically increased in month 3, and thereafter gradually reduced until month 9 when the quantity stabilized (age: *p* = 0.004; Figure 3A). The number of observed OTUs increased until month 10 after which it stabilized (*p* < 0.0001; Figure 3B). The Shannon and Simpson indexes were significantly lower in month 2 in comparison to the following months (age: *p* < 0.0001; Supplementary Figure S1). Both indexes reached the stable level in month 4 but experienced a drop around month 7, after which the levels gradually increased by month 10. The age explained 32% of the variation observed in the Bray-Curtis distances of ciliate protozoa (*R*^2^: 0.321, *p* = 0.001; Figure 3C). The first axis of principal coordinate analysis separated the communities by age, months 2–7 being separated from the communities of following months, while the second axis separated the T-group communities at month 2 from the other communities. The abundance of 12 species was affected by age (Figure 3D; Supplementary Table S5). The relative abundance of six protozoa species reduced with age, *Eremoplastron dilobum*, *Epidinium caudatum,* and *Isotricha* sp. being among the most abundant of these. The abundance of *Isotricha* sp. reduced in both groups by month 4, while *Eremoplastron dilobum* reduced in both groups by month 8, and *Epidinium caudatum* by month 9. The abundance of six species increased toward adulthood, of which *Isotricha prostoma* became the dominant species after month 8, *Entodinium longinucleatum* increased in abundance between months 3 and 11, while *Dasytricha ruminantium* and other rarer species became more abundant only after month 9.

The Random Forest model of ciliate protozoa community predicted a significantly faster community maturation of in the T-group in month 2, being 1.5 months ahead of the maturity of C-group. Thereafter, the predicted ages of two groups became similar (Supplementary Figure S3A). When looking at the approximate Shapley values for the T-group in month 2, the low abundance of OTUs affiliated with *Ostracodinium dentatum*, *Polyplastron multivesiculatum*, *Isotricha prostoma*, *Entodinium longinucleatum*, and *Isotricha intestinalis* was among the taxa that reduced the age prediction, while the low abundance of OTUs affiliated with *Eremoplastron dilobum* increased the predicted age (Supplementary Figure S3B). Looking at all the 31-maturation associated ciliate protozoa OTUs (Supplementary Figure S3C) taxa such as *Metadinium medium*, *Polyplastron multivesiculatum*, *Epidinium* spp., and *Isotricha* spp. were getting more abundant at later ages, while *Eremoplastron dilobum, Entodinium* sp., and *E. nagellum* were more abundant when the animals were young.

### The anaerobic fungi community was unaffected by treatment

**Figure 4:**
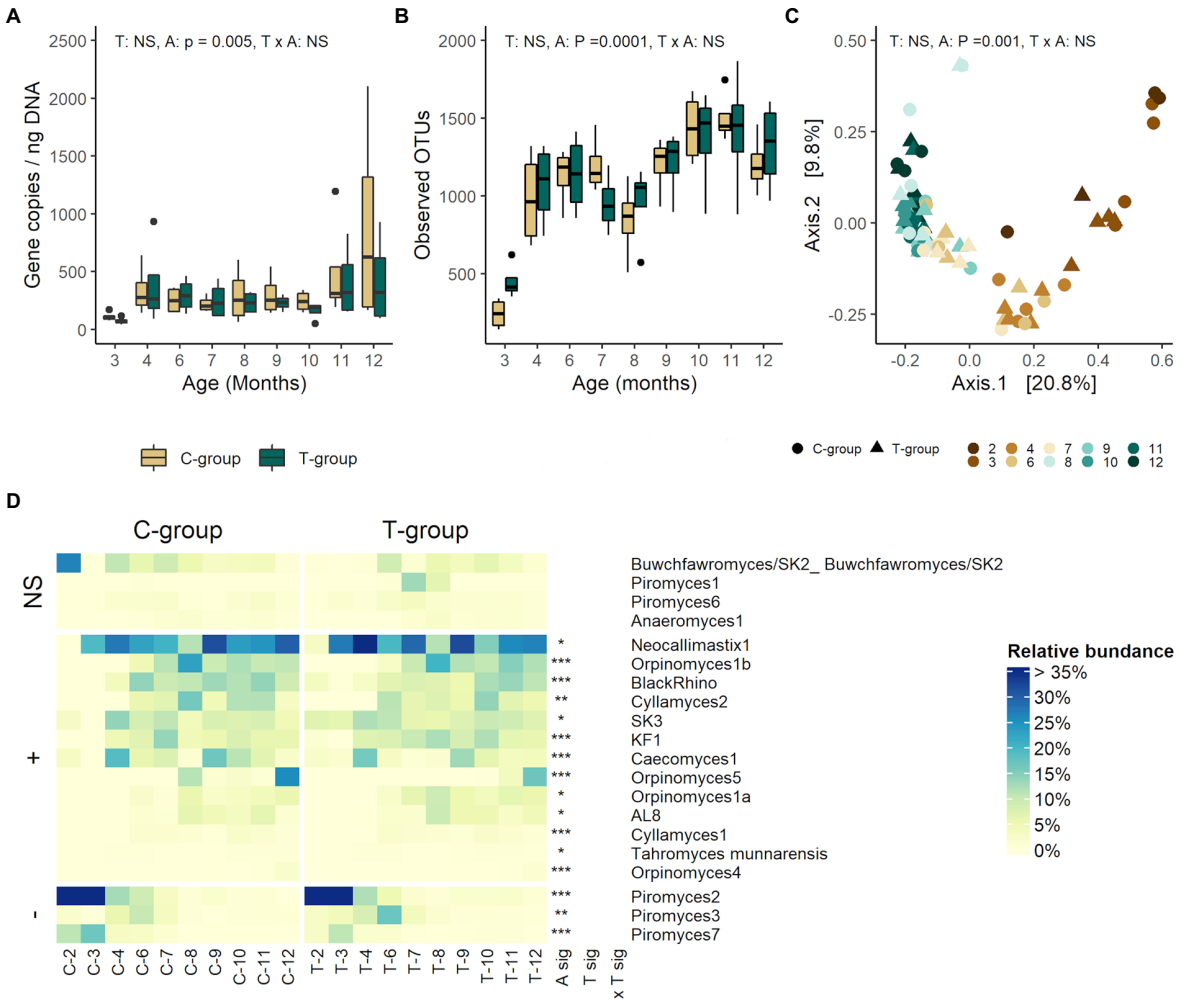
The rumen anaerobic fungi community in treatment (T-group) and control (C-group) heifers at 2–12 months of age. **(A)** The rumen anaerobic fungi ITS1 area copy numbers, **(B)** the number of observed OTUs, **(C)** principal coordinate analysis visualization of Bray-Curtis community distances, and **(D)** the relative abundances of protozoa species. Only taxa with statistical significances are shown. The taxa are grouped by increasing (+) or decreasing (−) trend with time, and with no significant effects (NS). Statistical significance of Treatment (T-sig), Age (A-sig), and Treatment × Age interaction (T × A sig): *p* < 0.05: *, *p* < 0.01: **, *p* < 0.001: ***.

The treatment had no effect on the anaerobic fungi ITS1 gene copy numbers (treatment: *p* = 0.385; Figure 4A), OTU richness (*p* = 0.626; Figure 4B), alpha diversity (Supplementary Figure S1), or beta diversity (*R*^2^ = 0.012, *p* = 0.19; Figure 4C). There were no statistically significant differences in relative abundance of any of the anaerobic fungi species between the groups (Figure 4D)

### The anaerobic fungi community established late

Evidenced by the low fungal ITS1 gene copy numbers, the communities were still established in both groups between months 2–3 (age: *p* = 0.005; Figure 4A). Anaerobic fungi were detected in month 2 in only one sample in the T-group, and three samples in the C-group. Between months 4–10, the copy numbers remained at a stable level, but again increased after month 11. The OTU richness increased significantly with age, reaching its peak in month 11, but temporarily declined between months 7–8 (*p* < 0.0001; Figure 4B). The similar stepwise increase was observed in Shannon index continuing up to 10 months (age: *p* < 0.0001; Supplementary Figure S1). Age explained 35% of variation between fungal community distances (*R*^2^ = 0.355, *p* = 0.001; Figure 4C). The PCoA visualization of Bray-Curtis distances separated the communities by age, where the communities in month 2–3, month 4–7, and month 9–12 were more similar to each other. The species-level taxonomic composition showed that in months 2 and 3, *Piromyces* 2 and *Piromyces* 7 dominated the community in both groups (Figure 4D; Supplementary Table S6). The anaerobic fungal richness started increasing in month 3, and the gradual development of fungal community structure continued until month 10, after which it stabilized (all pairwise: *p* < 0.05). From month 3 onward, *Piromyces* 2 and *Piromyces* 7 reduced in abundance in both groups and were replaced by *Neocallimastix* 1 as the dominant species accompanied by *Caecomyces* 1 and SK3 in month 4. Both community diversity and evenness further increased with increasing abundances of, e.g., *Cyllamyces* 2, *Piromyces* 3 and *BlackRhino* in month 6, KF1 and *Orpinomyces* 1b in month 7, and *Orpinomyces* 5, *Orpinomyces* 1a, and AL8 in month 8. By month 11, the evenness and diversity of the fungal community had reached its peak, as 11 species were observed with a relative abundance of more than 1.5%.

Random Forest model, based on the 45 age-related OTUs, indicated similar maturities in both groups (Supplementary Figure S4A). Similar to C-group, the Shapley values of the T-group’s model prediction from 2 to 12 months of age showed that higher abundances of *Neocallimastix* 1, *Orpinomyces 1a*, *Caecomyces* 1, *BlackRhino,* and *Orpinomyces* 1b were associated with a more mature community, whereas *Piromyces* 7, *Tahromyces munnarensis*, and *Caecomyces* 1 were associated with young community (Supplementary Figures S4B,C).

### Rumen function and growth of heifers

There were no significant differences between the groups in the weight of the heifers (treatment: *p* = 0.627, interaction: *p* = 0.992; Supplementary Table S7). The T-group gained on average 267 ± 9 kg and C-group 272 ± 9 kg during the 1^st^ year period.

The total VFA concentration decreased significantly with age (age: *p* < 0.019), and acetate proportion increased, while the proportions of all other individual VFAs reduced or had monthly fluctuation (Supplementary Figure S5). Butyrate (interaction: *p* = 0.04) was significantly affected by the treatment. The butyrate proportion was at its highest in month 3, with numerically higher proportion in the T-group.

### Host genetic effect on the microbial community similarity

**Figure 5:**
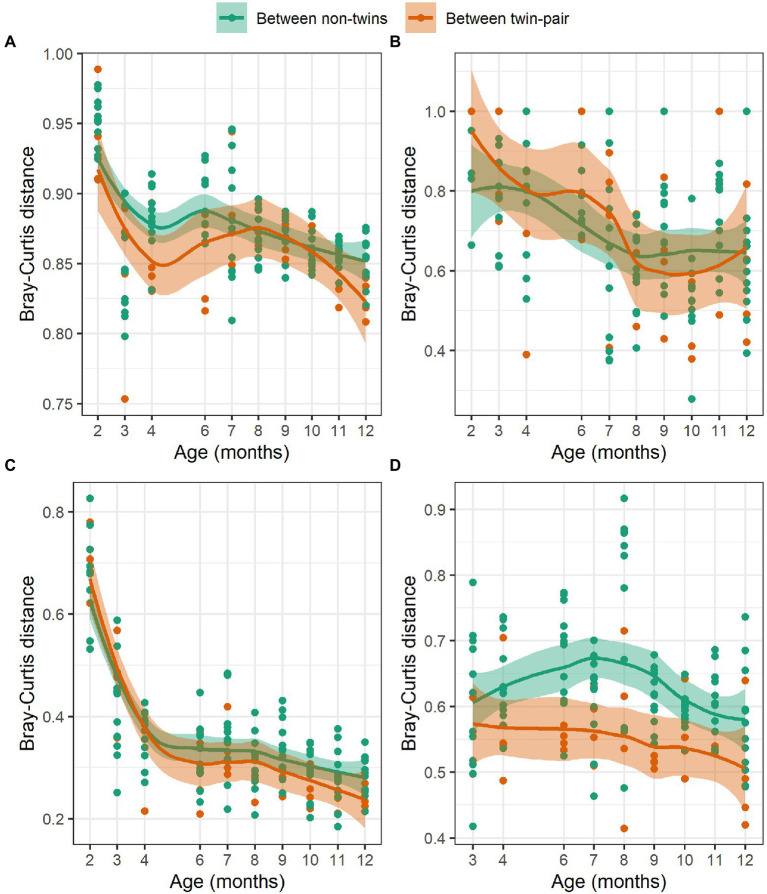
The Hellinger transformed Bray-Curtis distances of **(A)** bacteria, **(B)** archaea, **(C)** ciliate protozoa, and **(D)** anaerobic fungi communities between twin pairs (red) and between non-related heifers (green) at 2–12 months of age.

To assess if the host genetics influences the rumen microbial community composition, we compared Bray-Curtis distances within twin-pairs against non-sibling animals (Figure 5). We hypothesized that inoculation will be the major factor influencing rumen microbiota differences between the animals right after the weaning. If host genetics has an impact, the community distances between the twins will be smaller as compared to the distances between non-related animals during rumen maturation. Bray-Curtis distances reduced over time and were smaller between the twins than between the non-related animals at the end of the 12-month period for bacteria (Figure 5A), ciliate protozoa (Figure 5C) and anaerobic fungi (Figure 5D). A similar trend was not observed for archaea (Figure 5B).

### Rumen microbiome and rumen function during the first lactation

There were no significant differences between the groups in the total quantity of rumen bacteria, archaea, ciliate protozoa, or anaerobic fungi (Supplementary Table S8), or ASV/OTU alpha diversity (Supplementary Table S8) during the first lactation period. The Bray-Curtis distances of bacteria and fungi did not significantly differ between the groups (Adonis *p* > 0.05; Supplementary Figure S6), while differences in archaea and ciliate protozoa community structure between the groups were significant (Adonis *p <* 0.05). From the total of 83 bacterial genera observed (Supplementary Table S9), the T-group had higher relative abundances of unidentified genera affiliated with *Lachnospiraceae* (*p* = 0.025), *Lachnospiraceae NK3A20* group (*p* = 0.03), *Lachnospiraceae XPB1014* group (*p* = 0.066) and (*Eubacterium*) *xylanophilum* group (*p* = 0.067) as well as lower abundance of unidentified genera affiliated with *Prevotellaceae* (*p* = 0.058). *Methanobrevibacter gottschalkii*, *Mbb. ruminantium*, *Methanosphaera ISO3-F5,* and 6 less abundant (<5% relative abundance) archaeal species were observed within the dataset, but no significant differences between the groups were detected (Supplementary Table S10). In both groups, ciliate protozoa were represented by 16 species with *Entodinium* spp. and *Entodinium furca monolobum* being predominant, although two C-group heifers had high abundance of *Epidinium caudatum* (Supplementary Table S11). Only *Diplodinium dentatum* was more abundant in the C-group (*p* = 0.037), while *Entodinium longinucleatum* was significantly more abundant in the T-group (*p* = 0.043). The fungal community was represented by 10 genera in both groups (Supplementary Table S12). *Neocallimastix* and *Buwchfawromyces/SK2* were predominant fungi, but there was a high between-sample variation.

No significant differences between the groups were observed in total VFA concentration or in any other individual VFA proportions (Supplementary Table S13).

### Early life modulation effect on cows’ production performance

**Figure 6:**
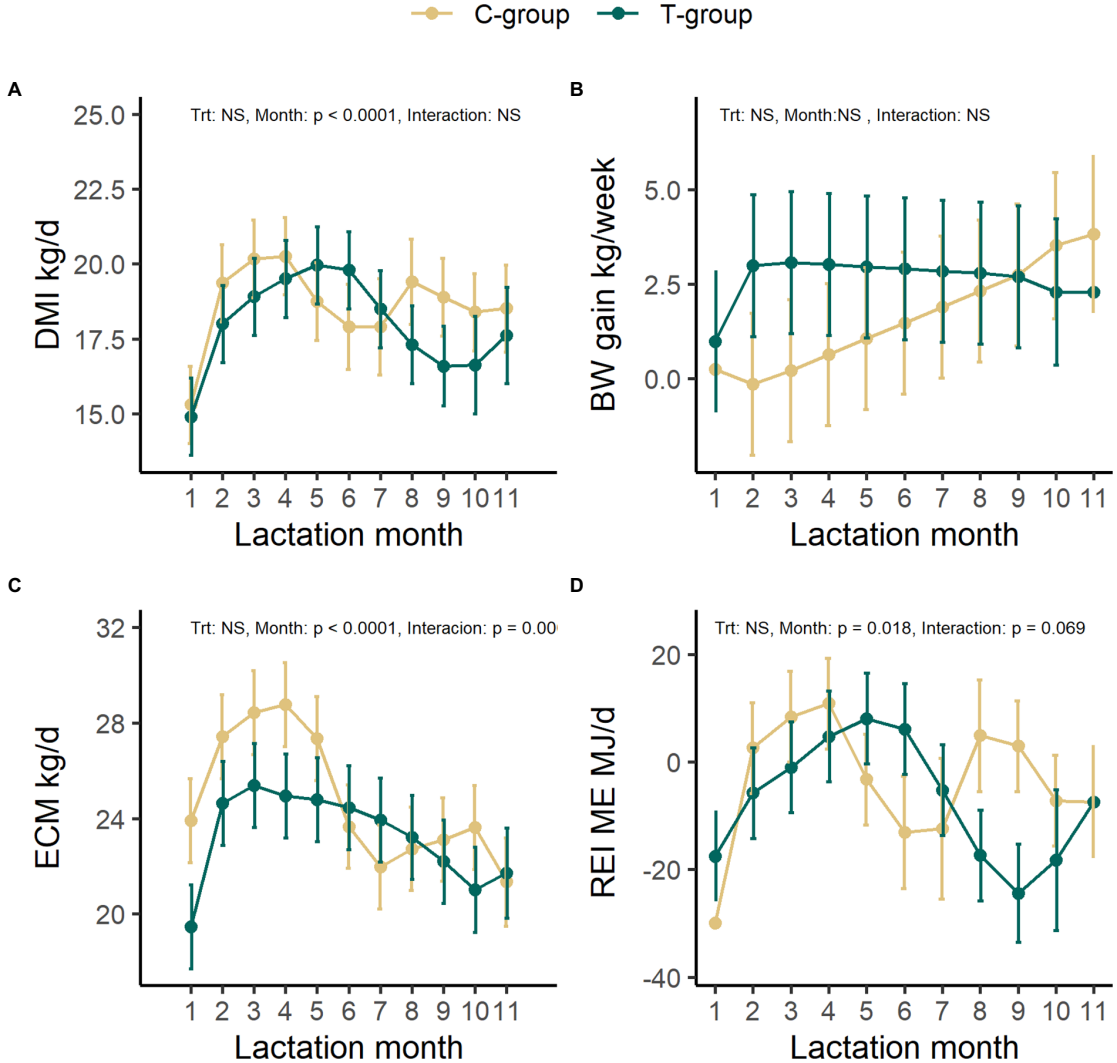
The average feed dry matter intake (DMI, kg/day) **(A)**, average weekly body weight gain **(B)**, energy-corrected milk yield (ECM, kg/day) **(C)**, and residual energy intake (REI) **(D)** in treatment (T-group) and control (C-group) cows during the 1^st^ lactation period. Data is presented in least square means ± standard error.

During the first lactation, no significant differences were observed in the DMI between the groups (Figure 6A). The C-group ate on average 18.6 kg DM/d corresponding to 204 ± 10 ME MJ/d, while the T-group ate on average 18.0 kg DM/d corresponding to 197 ± 10 ME MJ/d. The DMI increased until month 4 in the C-group and month 5 in the T-group after which it started to reduce (month: *p* < 0.0001).

There was no significant difference in the average BW between the groups, although the body weight of T-group was numerically lower (523 ± 26 kg) in comparison to C-group (563 ± 26 kg) at the beginning of lactation. The BW increased from 543 ± 18 kg in the first lactation month to 628 ± 18 kg in the end of the lactation period (Month: *p* < 0.0001) (Supplementary Table S14). Due to high between-animal variation, there were no significant differences between the groups or lactation months in the weekly weight gain of the cows (Figure 6B). The cows from T-group gained on average 2.6 ± 1.6 kg/week and from the C-group 1.6 ± 1.6 kg/week during the 1^st^ lactation. However, the T-group maintained a more even weight gain through the whole lactation as compared with the C-group, while C-group tended to lose weight during the second lactation month.

The average daily ECM yield was 24.8 ± 1.6 kg for C-and 23.3 ± 1.6 kg for the T-group and varied with month (treatment: *p* = 0.504, Month: *p* < 0.0001, interaction: *p* = 0.0009; Figure 6C). The peak in ECM yield in the C-group was observed in month 4 (28.8 ± 1.8 kg), but from 5 months onward the ECM rapidly diminished. In the T-group the ECM reached the highest yield (25.4 ± 1.8 kg) at 3 months after calving and remained more stable throughout the remaining lactation period. There were no significant differences in milk lactose, fat, or protein percent between the groups (Supplementary Table S15). The overall protein production (C-group:0.88 ± 0.05 kg; T-group: 0.83 ± 0.05 kg, *p* = 0.498), lactose production (C-group:1.01 ± 0.06 kg; T-group: 0.96 ± 0.06 kg, *p* = 0.595), and fat production (C-group:1.03 ± 0.08 kg; T-group: 0.95 ± 0.08 kg, *p* = 0.494) did not differ between groups (Supplementary Table S15). However, numerically higher milk yield in the C-group during the early lactation resulted into a numerically higher lactose (treatment × month: *p* = 0.002), protein (treatment × month: *p* = 0.001), and fat production (treatment × month: *p* = 0.001) during the first 5 months, in comparison to later months, while the production in T-group increased after first month, and remained at the same level thereafter. Treatment tended to affect the milk somatic cell count (SCC; treatment: *p* = 0.081; Supplementary Figure S6). Throughout the first lactation period the C-group had a numerically higher SCC (36 ± 1.1 × 1,000/ml) in comparison to the T-group (14 ± 1.1 × 1,000/ml).

The REI was affected by the stage of lactation in both groups (*p* = 0.018) but tended to have a different pattern of cycles of low and high REI between the group (interaction: *p* = 0.069; Figure 6D). Numerically, the mean REI was negative in the beginning and at the end of lactation in both C-and T-groups. The C-group had numerically lowest REI (−30 ± 8 ME MJ/d) in 1^st^ lactation month, while the T-group in 9^th^ month (−24 ± ME MJ/d). The shifts from negative to positive REI were delayed in the T-group, occurring *ca.* 2 months later in comparison to C-group. The T-group showed a longer period of negative REI in the early lactation, and lower and longer negative REI in the late lactation in comparison to C-group, while the C-group returned to negative REI earlier in mid-lactation and experienced a second positive REI peak in the late lactation.

The average methane production did not differ between the groups (C-group: 335 ± 14 g/d; T-group: 336 ± 16 g/d, LS mean ± SE, *p* = 0.951), but increased from 286.5 ± 19.9 g/d during the first lactation month to 373.3 ± 23.8 g/d in fifth lactation month (*p* = 0.02; Supplementary Table S14). After the first lactation month, the methane production in the C-group increased and remained stable, while the methane output in the T-group was more variable. There were no significant differences between the groups in the average methane yield (C-group: 18.1 ± 0.6, T-group: 17.4 ± 0.8 g/kg DMI; *p* = 0.378) or in the methane intensity (C-group: 13.7 ± 0.4, T-group: 15.0 ± 0.6 g/kg milk; *p* = 0.123). Both the methane yield and the methane production intensity maintained a similar level during 5 months measurement period (Month: *p* > 0.05; Supplementary Table S14).

## Discussion

In this study, we investigated the effect of rumen microbiota modulation during the pre-weaning period on the rumen microbiota maturation post-weaning and further elucidated its influence on the production performance of cows during their first lactation period. The present study builds upon our previous findings that inoculation with fresh rumen liquid can enhance the bacterial and archaeal community maturation before calves are weaned (Huuki et al., 2022). Here, we show that the effect of inoculation persisted post-weaning but gradually diminished, making the C-and T-groups similar in rumen microbiota structure before they reached 12 months of age. We also demonstrated that the inoculum treatment may affect the production performance during the 1^st^ lactation period in monozygotic twins, but a larger number of animals would be needed to reach significance.

### The transfer of microbes from inoculum donor into calves’ rumen before weaning

In our previous study (Huuki et al., 2022; Supplementary Tables S12–S14) we investigated the transfer of microbes between the donor cow and the recipient calves. We did this by comparing the number of shared OTUs between the donor and the core microbial community within the T-and C-groups at each sampling week during the pre-weaning period. The inoculum increased the OTU richness of the prokaryote and eukaryote core community in T-group starting from week 2. The number of OTUs shared with the donor increased with age in both groups, so that by weaning both groups shared all their prokaryote core OTUs with the donor. In a similar way, all anaerobic fungal and ciliate protozoan core OTUs detected within T-and C-groups at weaning were shared with the donor (Huuki et al., 2022). In the present study we no longer followed the donor community transfer, because, by weaning, all the core community microbes were shared with the donor. Instead, we focused on the further microbiota development with age, and on the community differences between the groups.

### Microbial community development until 1  year of age

#### Bacteria

The rumen modulation during the pre-weaning period influenced the bacterial community maturation for at least 2 months after the treatment. Based on the age-determinant taxa, the T-group calves were predicted to be older in comparison to the C-group until around month 4, after which the difference in predicted age was no longer apparent. The more mature community in the T-group was associated with increased abundance of *Prevotella*, *Christensenellaceae R-7* group, and *Lachnospiraceae* NK3A20 group as well as higher abundance of *Pseudobutyrivibrio*, and *Clostridiales* bacterium. Interestingly, ASVs affiliated with these taxa were significant contributors to maturation also in grazing yaks (Guo et al., 2020). Our observations are in line with the results of de Barbieri et al. (2015a), where inoculation of lambs with rumen liquid from ewes fed either coconut oil or rumen protected fat induced changes in the bacterial community lasting up to 3 months after the treatment. Similarly, different diets induced significant differences in the bacteria community composition in pre-weaned calves, but later these differences greatly diminished or disappeared (Dill-McFarland et al., 2019). The microbiome composition in the rumen is controlled by deterministic effects of both diets, in terms of available substrates for the function of microbes, and age of the animal, in terms of anatomical and physiological constraints, but also stochasticity that especially may affect the microbial communities in early life (Furman et al., 2020). It is possible that the plasticity of the rumen microbial community allowed the microbes obtained through the pre-weaning inoculation to be still active in microbial interactions, but with shared diet and environment, the communities gradually became more alike.

We saw major changes occurring in the bacterial community composition right after weaning (2–3 months) and continuing until 7 months of age, after which only minor changes were observed. The taxonomical changes around weaning were related to the decreasing abundance of Proteobacteria, such as *Succinivibrio* or *Succinivibrionaceae* spp., and several Bacteroidota genera, such as *Alloprevotella* and *Prevotellaceae* spp., but also shifts in the taxonomic composition of Firmicutes. Bacteroidota are known to be abundant in the pre-weaned calves (Jami et al., 2013; Rey et al., 2014), but major shifts within Bacteroidota occur by weaning due to increasing dominance of *Prevotellaceae* family that is linked to increasing diet fiber content (Rey et al., 2014; Furman et al., 2020). Similarly, the Proteobacteria are more abundant in young calves (Jami et al., 2013), and *Succinivibrionaceae* has been observed to decrease around weaning, which was speculated to be associated with increasing fiber content in the diet and increasing abundance of *Methanobacteriaceae* family archaea (Furman et al., 2020). On the other hand, our results showed, that the bacterial community continued to develop until month 7, mainly due to shifts in abundances of several Firmicutes genera. Changes in some rare taxa were observed even up to 10 months of age. Firmicutes contain many important cellulolytic taxa, that have a role in fiber degradation (Deusch et al., 2017; Terry et al., 2019). The observed shifts in the Firmicutes taxonomical composition with age could be an adjustment to the changing diet or could be an indication of changes in the rumen microbial interactions, as an increase in abundance of, e.g., *Lachnospiraceae* family has been previously found without changes in the diet (Furman et al., 2020). Our results on the timeline of the rumen maturation agree with some of the few studies that have followed the natural progress of rumen bacteria community development until adulthood, which showed that rumen core community establishes by 5 months of age, but few and less persistent changes related to other taxa continued to occur until adulthood (Furman et al., 2020).

#### Archaea

The archaea community post-weaning was not affected by treatment, and experienced small taxonomical changes until month 6. These changes were related to *Methanosphaera* spp. being more abundant in younger heifers but being replaced by *Methanobrevibacter gottchanlkii* as the dominant species after month 3. *Methanobacterium alkaliphilum*, *Methanomassiliicoccaceae* Group3b sp., and *Mmc*. Group8 sp. were late arrivals and increased in abundance after month 8. The increasing taxonomical diversity of archaea is possibly linked to the rumen metabolite diversity, which increases with age due to the more complex microbial functional interactions. Our observations are in line with Dias et al. (2017) who demonstrated that *Methanosphaera* was dominating in the pre-weaning calves fed milk and concentrate diet, while *Mbb. gottschalkii* dominated in calves receiving milk. While *Mbb. gottschalkii* is a hydrogenotrophic methanogen (Miller and Lin, 2002), *Methanosphaera* uses methanol for methanogenesis (Miller and Wolin, 1985). Methanol is produced in pectin degradation, with *Prevotella* spp. being regarded as the main producer of methanol in the rumen (Kelly et al., 2019). *Methanomassiliicoccaceae* spp. can also utilize methanol as an electron acceptor, but unlike *Methanosphaera*, they are also able to utilize methylamines (Poulsen et al., 2013; Söllinger et al., 2018), and have advantage in competition for substrates with *Methanosphaera. Methanobacterium alkaliphilum,* on the other hand, is known to tolerate high pH (Worakit, 1986). The greater abundance of *Methanobacterium alkaliphilum* during the last months could be related to potential changes in the rumen pH caused by changes in feed/silage quality, but unfortunately, rumen pH was not measured to confirm this hypothesis.

#### Ciliate protozoa

The initial housing conditions for transmission of ciliate protozoa differed between the two groups, resulting in *Eremoplastron dilobum* dominating the C-group, whereas *Isotricha* sp. and *Epidinium caudatum* being predominant in the T-group by the end of the pre-weaning period (Huuki et al., 2022). While the T-group received ciliate protozoa with rumen liquid inoculum, and in all the T-group heifers protozoa were observed already in month 1 (Huuki et al., 2022), majority of the C-group calves remained defaunated and acquired ciliate protozoa only by month 3, when they were moved to a group pen. Therefore, the T-group community had more time to establish, and the treatment continued to enhance the maturation of ciliate protozoa community until animals reached 4 months of age. However, from month 3 onward, the communities of both groups started to become more alike, and the C-group quickly caught-up in maturation. The ciliate protozoa are transmitted through a contact with conspecifics and contaminated environment (Bird et al., 2010). The translocation of heifers into a different barn, and the housing of calves from both groups inside the same group pen may have caused exchange of different ciliate protozoan taxa, and shifts in the relative abundance of species, that resulted in similar community composition between the groups at the age of 7–8 months, dominated by *Isotricha prostoma*. While, the ciliate protozoa have been intensively studied in terms of methane mitigation and protein metabolism, recent research on the long-term community changes is sparse. Despite ciliate protozoa being robust in their group persistence (Eadie, 1962a,b), it looks that C-group, with different taxa abundant at month 2, was still flexible for changing ciliate community composition.

#### Anaerobic fungi

In our previous study, we demonstrated that the adult animal rumen liquid inoculum, provided to the calves during the pre-weaning period, did not stimulate earlier anaerobic fungi establishment and rather had the opposite effect (Huuki et al., 2022). By following rumen microbial community development post-weaning, we demonstrated that the quantity of rumen fungi remained low until month 4, and that the development of the anaerobic fungi community structure and the increase in richness was gradual and continued at least until month 10, after which it stabilized.

In our study, the pre-weaning treatment did not induce fungal community composition differences between the groups in any of the community diversity measures post-weaning. Dill-McFarland et al. (2019) found that the pre-weaning diet affected the fungal community later in life in cows, and Belanche et al. (2019a) showed that natural rearing with lambs enhanced the fungal community establishment and had long-term impact on fungal richness when lambs were grazing. Together these observations suggest that the diet determines the fungal community composition, and the establishment of anaerobic fungi in the rumen cannot be aided by the pre-weaning inoculation with rumen fluid from an adult cow. It is possible that in the case of anaerobic fungi, the best time window for modulation is only after the weaning, when the rumen environment can maintain the community and the establishment properly begins.

The anaerobic fungi taxonomical composition continued to develop at least until month 10. During milk feeding period, *Caecomyces* and SK3 were the most abundant taxa (Huuki et al., 2022). The diet before weaning is rich in fast fermentable carbohydrates, and high levels of fermentation products could have an inhibiting effect on anaerobic fungi (Joblin and Naylor, 1993). As the solid feed intake increased toward the weaning, *Caecomyces* and SK3 were gradually replaced by *Buwchfawromyces*/SK2, and *Piromyces 2* and 7 became dominant around month 3. These *Piromyces* species have not yet been cultured and their functional properties are not yet characterized. However, some *Piromyces* strains are known to possess very high hydrolytic activities in comparison to other anaerobic fungi (Tripathi et al., 2007), which could give them advantage in competition for increasing substrates during the early stages of rumen development. From month 4 onward, the *Piromyces* spp. were replaced by *Neocallimastix* 1, which retained the dominant status until the end of the experiment.

Secondly, the taxonomic richness and evenness of the fungal community increased with age. While at weaning the *Piromyces* 2 abundance reached 62%, and only five other taxa were detected at a relative abundance >1%, the fungal taxonomic richness and evenness greatly increased between months 4 and 10. This period in rumen anaerobic fungi development was described by fluctuation among various fungal species, including *Orpinomyces* spp. It is likely, that the increasing solid feed intake, the more stable rumen environment, and more developed rumen microbial ecosystem supported the establishment of increasing number of fungal species, but also induced competition between the species, which was seen in increased evenness estimate. Competition between *Orpinomyces* and *Neocallimastix* has been previously proposed by (Kittelmann et al., 2012) as they seem to have structurally similar cellulase and xylanase enzymes (Li et al., 1997). The taxonomic composition of rumen anaerobic fungi has been found to vary with the diet in adult animals, e.g., the *Neocallimastix* being more dominant in high concentrate-to-forage ratio diet (Tapio et al., 2017).

### Rumen volatile fatty acids until 1  year of age

The age-dependent patterns of rumen VFA likely reflected the changes in the feeding regime and quality of the feed. The proportion of acetate had a slight increase, while the propionate and butyrate proportions reduced with age, most significant changes occurring after weaning at month 3. A high concentrate diet has been shown to increase the production of butyrate and propionate due to breakdown of carbohydrates into sugars by amylolytic bacteria. The hexoses are further broken down to pyruvate, which is used to produce butyrate, and propionate through intermediate metabolites such as lactate and succinate (Zhang et al., 2020). We saw a reduction in the relative abundance of several sugar utilizing, succinate, and/or lactate-producing taxa, e.g., *Selenomonas* (Silley and Armstrong, 1985), *Succinivibrio* (Bryant and Small, 1956), *Alloprevotella* (Downes et al., 2013), *Blautia* (Liu et al., 2021), and, e.g., in propionate producing taxa such as *Succiniclasticum* (Van Gylswyk, 1995). Therefore, it is tempting to speculate that the changes in diet around weaning may have contributed to the reduction of fermentation intermediates such as lactate and succinate and led to the decrease of propionate and butyrate proportions. This is further supported by previous studies, which have reported higher concentrations of fermentation intermediates in calves, as well as a decrease in propionate and increase in acetate proportions in response to changing from milk diet toward fiber and starch diet (Dill-McFarland et al., 2019), or decreasing acetate to propionate ratio and butyrate concentration (Anderson et al., 1987). Dill-McFarland et al. (2019) suggested that the decrease in propionate is related to increased maturation of gastrointestinal tract, as propionate is the main substrate of gluconeogenesis in ruminants.

Despite some differences in microbial community composition between the T-and C-group, we observed only minor differences in the VFA proportions between the groups, which was likely due to functional plasticity within the rumen (Taxis et al., 2015). In comparison to C-group, the T-group tended to have higher proportion of butyrate around month 3. Many of the taxa, identified by Random Forest to be related to more mature community in T-group around the same time, are known to include butyrate-producing species, e.g., *Christensenellaceae* (Morotomi et al., 2012) and *Pseudobutyrivibrio* (Van Gylswyk et al., 1996). The overall higher abundance of butyrate-producing taxa may have contributed to the numerically higher butyrate proportion in T-group. Moreover, the ciliate protozoa quantity peaked in month 3 in both groups, being slightly higher in T-group in comparison to the C-group. The ciliate protozoa have been shown to contribute to the VFA production to a varying degree, depending on the protozoa community structure (Solomon et al., 2022). Also, it has been shown that the defaunation of ruminants from ciliate protozoa may have a negative effect on the butyrate production (Newbold et al., 2015). We therefore speculate, that the ciliate protozoa contributed to the butyrate concentration peak around month 3 and can partially explain the numerically higher butyrate proportion in T-group.

### Host genetics effect on rumen microbiome

The host genetics is known to affect the ruminant microbiota (Li F. et al., 2019; Wallace et al., 2019). An increasing body of evidence suggests that the transfer of bacteria and archaea from the mother to fetus starts already in the uterus (Guzman et al., 2020; Husso et al., 2021) and microbiome between the twins in humans (Turnbaugh et al., 2009; Goodrich et al., 2014) and bovine (Mayer et al., 2012) is more similar than between unrelated individuals. To minimize the variation caused by the host genetics in the present study, we used monozygotic twin calves, produced by embryo splitting. Due to inoculation treatment during the pre-weaning period, we observed differences in the rumen microbial community composition between the C-and T-groups (Huuki et al., 2022). However, we hypothesized that if the hosts’ genetics affects its own microbiome development, the impact will be visible after rumen modulation is over and animals are left to develop until adulthood. In this study, we observed that the treatment effect gradually diminished and the microbial communities in all individuals became more alike with age. However, apart from the archaea, the Bray-Curtis distances in all other domains became smaller with age between the twin pairs in comparison to distances between the non-siblings. The impact of host genetics on ciliate protozoa and fungi has not been extensively studied, and currently existing data suggest that the rumen eukaryome is not considered to be highly hereditary (Gonzalez-Recio et al., 2018; Wallace et al., 2019; Martínez-Álvaro et al., 2022). Yet, we found that the communities tended to be more similar within the twin-pairs in comparison to non-siblings. It is tempting to speculate that this greater similarity could be a result of host genetic impact on own pro-and eukaryome development, that became apparent when the effect of inoculation diminished. However, a study with larger number of animals is needed to confirm our preliminary findings.

### First lactation period animal performance

We observed some differences in production parameters between the T-and C-groups during their 1^st^ lactation period. During the first four lactation months, the C-group maintained numerically higher milk production in comparison to the T-group. The differences between the groups in early lactation could be due to numerically lower body weight in the T-group after parturition caused by slightly younger parturition age due to earlier insemination success. The lower body reserves at the start of the lactation period have been shown to direct the energy use toward growth and reduce the milk yield, because cows with higher BW are able to mobilize more energy from the body reserves, especially during early lactation (Mäntysaari et al., 1999; Mäntysaari and Mäntysaari, 2010). Between 2^nd^ and 4^th^ lactation month the C-group also started to compensate for the earlier body mass loss. In comparison, the T-group did not lose BW, although the BW gain temporarily slowed down during the first lactation month. The C-group had its highest efficiency (lowest REI) during 1^st^ month of lactation, while the T-group reached highest efficiency between 8^th^ and 10^th^ month of lactation, when the ECM and DMI gradually reduced, and more energy was used toward growth. These observations may suggest that the T-group partitioned more of the energy into growth than into milk production, while keeping a steady milk yield, whereas the C-group used more of the energy into milk production especially during the early lactation but had to invest more energy into growth in the late lactation. Previously, high, and low merit producers have been found to vary in partitioning of metabolizable energy into different energy sinks, and that there is genetic variation within these traits (Mehtiö et al., 2018).

There were no differences in milk composition between the groups, but differences were detected in SCC, with C-group having on average higher SCC in comparison to T-group throughout the whole lactation. Two of the C-group cows and one T-group cow were diagnosed with mastitis during their early lactation. The SCC of all these cows reduced to a healthy level (<150,000 cell/ml) after treatment. However, one C-group cow with mastitis and another C-group cow without clinical signs of mastitis retained SCC on average higher than 50,000 cells/ml throughout the experiment. A link between the rumen microbiome and clinical mastitis has been demonstrated in cows (Zhong et al., 2018, 2020) and in sheep (Boggio et al., 2021). Zhong et al. (2018) showed that the high SCC associated cows had a higher rumen bacterial diversity, changes in the bacterial taxonomical composition, a lower milk yield, and a lower milk lactose (%), but a higher milk fat and protein (%). No similar observations were made in our study, as the C-group had numerically higher milk yield in comparison to T-group, and the milk composition did not differ between the groups. The C-group showed signs of lower REI during the first lactation month in comparison to the T-group, which could have affected the higher SCC. It has been previously shown that the low REI often leads to greater negative energy balance, which may increase the risk of diseases (Becker et al., 2021).

Because the microbial community composition and rumen fermentation characteristics were similar between the groups, and the genetic background of individuals was identical, the production performance of the animals would be expected to be similar. Yet, subtle differences in production performance were observed. In general, the different seasons and the age of the cow can cause variation in the production performance experiments, which is normally resolved with a higher number of study subjects. Due to the limited number of animals in the present experiment, and the differences in calving times, we cannot rule out the possibility that these factors may have influenced the results. However, in the current experiment the genetic background, housing and dietary conditions were controlled, apart from pasture season. Therefore, the differences between the groups could be speculated to be affected by the rumen modulation treatment, especially during the first 5 lactation months, when none of the cows were on pasture and the diet was strictly controlled. We cannot rule out the possibility, that the rumen microbiome modulation during the pre-weaning period induced permanent changes in, e.g., calf’s metabolism (Li W. et al., 2019), immune development (Raabis et al., 2019), or intestinal development and/or function, that could indirectly impact the performance. Further studies are also needed to assess influence of the lower gut microbiome on the differences in the phenotypic characteristics, as some indications of changes caused by rumen liquid inoculum on colon microbiota exist (Yu et al., 2020) and in lambs’ differences in lower intestine microbiota have been associated with feed efficiency phenotype (Perea et al., 2017).

## Conclusion

Our results showed that the orally administered microbial inoculant enhanced the maturation of T-group bacteria and ciliate protozoa until month 4, and in both groups, the community development reached the adult animal stage between 8 and 9 months after birth. The pre-weaning treatment did not enhance the anaerobic fungi establishment, as the anaerobic fungi were the last domain that started efficient rumen colonization only post-weaning, and their gradual development to adult stage continued until month 10. The age-related changes in the archaea community were not as apparent as in bacteria, and the community tended to stabilize at 6 months of age. Some differences in production parameters were observed between the T-and C-groups during the 1^st^ lactation period. The T-group tended to have a more uniform energy-corrected milk yield and residual energy intake during the different phases of lactation, and had lower somatic cell count, as compared to the C-group animals. No differences in methane production were detected. However, further studies with a larger number of animals are needed to elucidate the mechanisms through which the early-life microbiome modulation influences the production performance in ruminants.

## Data availability statement

The datasets presented in this study can be found in online repositories. The names of the repository/repositories and accession number(s) can be found at: https://www.ncbi.nlm.nih.gov/, BioProject PRJNA713003 with corresponding BioSample Accessions SAMN28869958 - SAMN28870037.

## Ethics statement

The animal study was reviewed and approved by National Ethics Committee (ESAVI/5687/04.10.07/2017, Hämeenlinna, Finland).

## Author contributions

IT and JV: conceptualization and funding acquisition. HH, IT, PM, EN, and SA: methodology and data acquisition. HH and MT: data analysis. HH: writing original draft. IT, AV, MT, PM, SA, JV, and EN: writing, review, and editing. All authors contributed to the article and approved the submitted version.

## Funding

This project was funded by the European Union’s Horizon 2020 Research and Innovation program under grant agreement no. 818368 (MASTER). Production, intake, and methane data were provided by the A++ cow project funded by the Development Fund for Agriculture and Forest (Makera: 453/03.01.02/2018). The salary of Hanna Huuki was provided by the doctoral program of Sustainable Use of Renewable Resources, Department of Agricultural sciences, Faculty of Agriculture and Forestry, University of Helsinki, and personal grant “Suomi kasvaa ruoasta” (no. 20210072) funded by Oiva Kuusisto foundation.

## Conflict of interest

The authors declare that the research was conducted in the absence of any commercial or financial relationships that could be construed as a potential conflict of interest.

## Publisher’s note

All claims expressed in this article are solely those of the authors and do not necessarily represent those of their affiliated organizations, or those of the publisher, the editors and the reviewers. Any product that may be evaluated in this article, or claim that may be made by its manufacturer, is not guaranteed or endorsed by the publisher.
