# Supplementary material for: Long-term effects of early-life rumen microbiota modulation on dairy cow production performance and methane emissions
Source: Front Microbiol. 2022 Nov 8;13:983823. doi: 10.3389/fmicb.2022.983823 (PMC9679419; doi:10.3389/fmicb.2022.983823)
Supplement: Supplementary file 4 [file Data_Sheet_1.PDF]

Supplementary Material 1

1 Materials and methods

1.1 Quantification of bacterial communities

1.1.1 Preparation of standard curves

DNA extracted from mixed rumen sample was used as a template for standard curve preparation. The 16S ribosomal RNA gene (*rrs*) was amplified using 8-27F and 1512-1492R primers (**S** T**able S1**). The correct size of the amplicon (1492 bp) was verified with electrophoresis in a 2 % agarose gel, excised from the gel. The concentration of the amplicon was measured with Qubit 4 Fluorometer (Invitrogen by Thermo Fischer Scientific, Life Technologies Holdings Ltd, Singapore) with Qubit® dsDNA HS Assay (Invitrogen by Thermo Fischer Scientific, Life Technologies Corporation, Oregon, USA).

The number of copies was calculated with a formula,

$$\frac{6.02 \times 10^{23} \times DNA\ quantity}{660 \times amplicon\ size\ bp}$$

Where 6.02×10^{23} is the Avogadro constant, DNA quantity is the measured Qubit concentration in g/µl, 660 is the molecular mass of the nucleotide, and the amplicon size is the estimated number of nucleotides in the amplicon (1492 bp). The amplicon was then diluted to a concentration of 5×10^{9} and further diluted in a 10 base logarithmic series until 5×10^{2} copies/µl. From each dilution 2 µl of DNA was used in creating standard curve, giving a logarithmic scale of number of copies from 10^{3} to 10^{9}. Threshold cycle of each dilution was measured by amplifying the V4 area (279 bp) with a second set of primers UniF and UniR (**S Table S1**) in a Viia7 thermocycler (Applied Biosystems). Amplification reaction (10 µl) contained 1 x Power SYBR Green PCR master mix (Applied Biosystems by Thermo Fischer Scientific, Life Technologies Ltd, UK), 0.2 µM of each primer and 10 ng of DNA. The amplification was done in a Viia7 thermocycler (Applied Biosystems), with denaturation at 95 °C for 10 min, 40 cycles of denaturation at 95 °C for 15 s, annealing at 60 °C for 30 s, and extension at 72 °C for 30 s. A melt curve analysis was performed after amplification with denaturation at 95 °C for 15 s, annealing at 60 °C for 1 min and denaturation at 95 °C for 15 s, with a ramp increment of 0.4 °C. The effectiveness and the quality of the standard curve were checked, and information of the standard curve saved. Two points from the standard curve (10^{5} and 10^{7} copies per reaction) were aliquoted to smaller fractions and frozen to -80°C to be used as an inter-plate control.

1.1.2 Quantification of bacteria in rumen samples

The bacteria were quantified by amplifying the 16S rRNA gene area (279 bp) using primers UniF (3' - GTG STG CAY GGY TGT CGT CA – 5') and UniR (3' - ACG TCR TCC MCA CCT TCC TC - 5') (Maeda et al., 2003). Amplification reaction (10 µl) contained 1 x Power SYBR Green PCR master mix (Applied Biosystems by Thermo Fischer Scientific, Life Technologies Ltd, UK), 0.2 µM of each primer and 10 ng of DNA. The amplification was done in a Viia7 thermocycler (Applied Biosystems), with denaturation at 95 °C for 10 min, 40 cycles of denaturation at 95 °C for 15 s, annealing at 60 °C for 30 s, and extension at 72 °C for 30 s. A melt curve analysis was performed after amplification with

denaturation at 95 °C for 15 s, annealing at 60 °C for 1 min and denaturation at 95 °C for 15 s, with a ramp increment of 0.4 °C.

S Table S1. Primers used in the study.

Target organism		Primer	Sequence 5'-3'	Target area	Amplicon size bp	Reference
**16S standard preparation**						
Bacteria	F	F8-27	AGAGTTTGATCCTGGCTCAG	16S	1485	(Turnbaugh et al., 2009)
Bacteria	R	1512-1492R	GNTACCTTGTTACGACTT			
Archaea	F	344F	ACGGGGYGCAGCAGGCGCGA	16S	1062	(Ohene-Adjei et al., 2007)
Archaea	R	1406-1389R	ACGGGCGGTGTGTGCAAG			(Loy et al., 2002)
Ciliate protozoa	F	P.SSU-57F	CAYGTCTAAGTATAAATAACTAC	18S	1694	Sylvester et al. (2004)
Ciliate protozoa	R	P.SSU-1747R	CTCTAGGTGATWWGRTTTAC			
Fungi	F	Neo 18S For	AAT CCT TCG GAT TGG CT	ITS1	350-450	Edwards et al. (2008)
Fungi	R	Neo 5.8S Rev	CGA GAA CCA AGA GAT CCA			
**Quantitation with qPCR**						
Bacteria	F	UniF (1047)	GTG STG CAY GGY TGT CGT CA	V7	147	(Maeda et al., 2003)
Bacteria	R	UniR (1194)	ACG TCR TCC MCA CCT TCC TC			
Archaea	F	896-915F	AGGAATTGGCGGGGGAGCAC	V6-V8	510	(Stahl and Amann, 1991) (Ohene-Adjei et al., 2007)
Archaea	R	1406-1389R	ACGGGCGGTGTGTGCAAG			(Loy et al., 2002)
Ciliate protozoa	F	316F	GCTTTCGWTGGTAGTGTATT	18S	223	Sylvester et al. (2004)
Ciliate protozoa	R	539R	CTTGCCCTCYAATCGTWCT			
Fungi	F	FungiF	GAGGAAGTAAAAGTCGTAACAAGGTTTC	18S-ITS1	120	Denman & McSweeney (2006)
Fungi	R	FungiR	CAAATTCACAAAGGGTAGGATGATT			
**Amplicon sequencing**						
Bacteria & archaea	F	CapNexF (515F)	TCGTCGGCAGCGTCAGATGTGTATAAGAGACAGGTGCCAGCMGCCGCGGTAA	16S V4	290	(Caporaso et al., 2011)
Bacteria & archaea	R	CapNexR (806R)	GTCTCGTGGGCTCGGAGATGTGTATAAGAGACAGGGACTACHVGGGTWTCTAAT			
Ciliates	F	ILNA_316F	TCGTCGGCAGCGTCAGATGTGTATAAGAGACAGGCTTTCGWTGGTAGTGTATT	18S V3 (SR1)	225	(Sylvester et al., 2004)
Ciliates	R	ILNA_539R	GTCTCGTGGGCTCGGAGATGTGTATAAGAGACAGCTTGCCCTCYAATCGTWCT			
Fungi	F	ILNA_Neo18SF	TCGTCGGCAGCGTCAGATGTGTATAAGAGACAGAAT CCT TCG GAT TGG CT	ITS1	350-450	(Edwards et al., 2008)
Fungi	R	ILNA_Neo5.8SR	GTCTCGTGGGCTCGGAGATGTGTATAAGAGACAGCGA GAA CCA AGA GAT CCA			

2 References

Caporaso, J. G., Lauber, C. L., Walters, W. A., Berg-lyons, D., Lozupone, C. A., Turnbaugh, P. J., et al. (2011). Global patterns of 16S rRNA diversity at a depth of millions of sequences per sample. *PNAS* 108, 4516–4522. doi: 10.1073/pnas.1000080107/-/DCSupplemental.www.pnas.org/cgi/doi/10.1073/pnas.1000080107.

Edwards, J. E., Kingston-Smith, A. H., Jimenez, H. R., Huws, S. A., Skøt, K. P., Griffith, G. W., et al. (2008). Dynamics of initial colonization of nonconserved perennial ryegrass by anaerobic fungi in the bovine rumen. *FEMS Microbiology Ecology* 66, 537–545. doi: 10.1111/j.1574-6941.2008.00563.x.

Loy, A., Lehner, A., Lee, N., Adamczyk, J., Meier, H., Ernst, J., et al. (2002). Oligonucleotide Microarray for 16S rRNA Gene-Based Detection of All Recognized Lineages of Sulfate-Reducing Prokaryotes in the Environment. *Applied and Environmental Microbiology* 68, 5064–5081. doi: 10.1128/AEM.68.10.5064.

Maeda, H., Fujimoto, C., Haruki, Y., and Maeda, T. (2003). Quantitative real-time PCR using TaqMan and SYBR Green for Actinobacillus actinomycetemcomitans, Porphyromonas gingivalis, Prevotella intermedia, tetQ gene and total bacteria. *FEMS Immunology and Medical Microbiology* 39, 81–86. doi: 10.1016/S0928-8244(03)00224-4.

Ohene-Adjei, S., Teather, R. M., Ivan, M., and Forster, R. J. (2007). Postinoculation protozoan establishment and association patterns of methanogenic archaea in the ovine rumen. *Applied and Environmental Microbiology* 73, 4609–4618. doi: 10.1128/AEM.02687-06.

Stahl, D., and Amann, R. (1991). “Development and application of nucleic acid probes.,” in *Nucleic Acids Techniques in Bacterial Systematics*, eds. E. Stackebrandt and M. Goodfellow (Chichester: John Wiley & Sons,), 205–248.

Sylvester, J. T., Karnati, S. K. R., Yu, Z., Morrison, M., and Firkins, J. L. (2004). Development of an Assay to Quantify Rumen Ciliate Protozoal Biomass in Cows Using Real-Time PCR. *Nutritional Methodology* 134, 3378–3384. doi: 10.1093/jn/134.12.3378.

Turnbaugh, P. J., Hamady, M., Yatsunenko, T., Cantarel, B. L., Duncan, A., Ley, R. E., et al. (2009). A core gut microbiome in obese and lean twins. *Nature* 457, 480–484. doi: 10.1038/nature07540.
