# Supplementary material for: Long-term effects of early-life rumen microbiota modulation on dairy cow production performance and methane emissions
Source: Front Microbiol. 2022 Nov 8;13:983823. doi: 10.3389/fmicb.2022.983823 (PMC9679419; doi:10.3389/fmicb.2022.983823)
Supplement: Supplementary file 5 [file Data_Sheet_2.pdf]

Supplementary Material 2

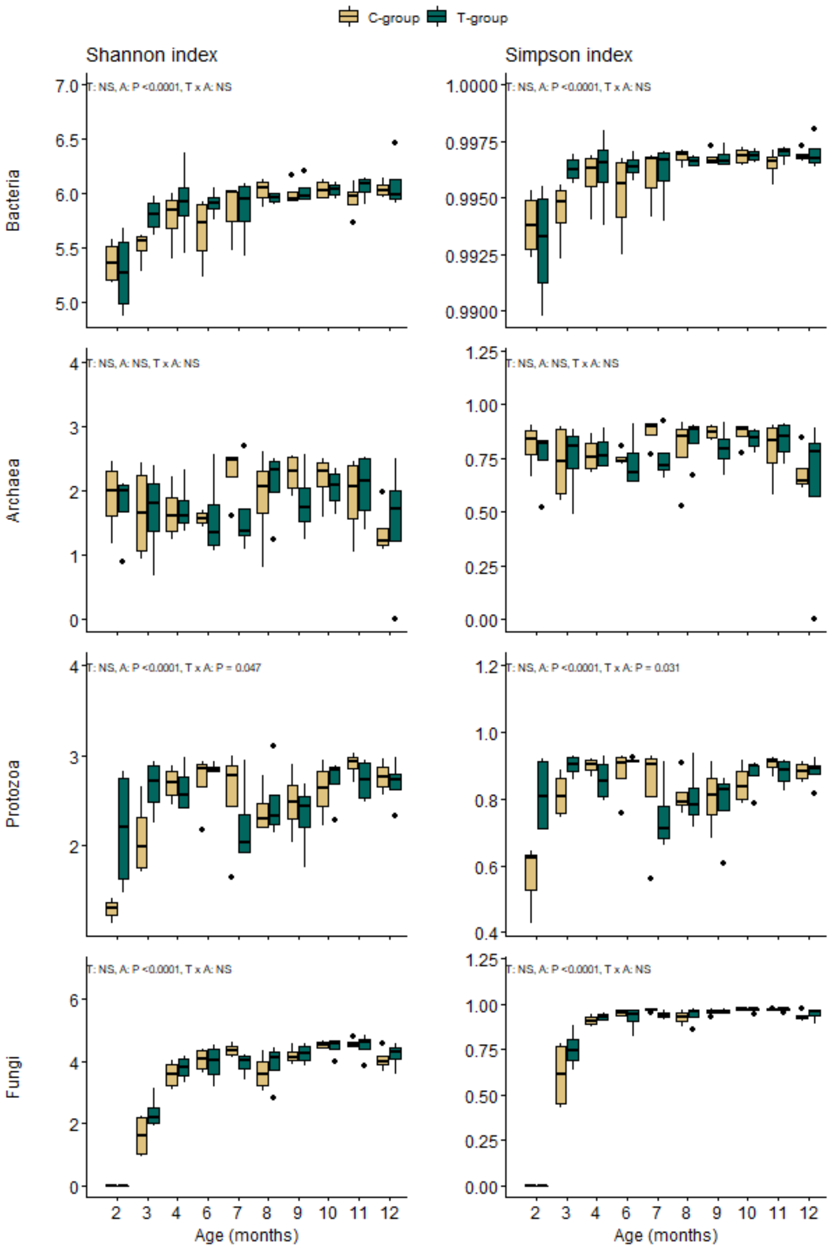

Figure S1. Shannon and Simpson indexes of rumen bacteria, archaea, protozoa, and anaerobic fungi in treatment (T-group) and control group (C-group) heifers at 2-12 months of age.

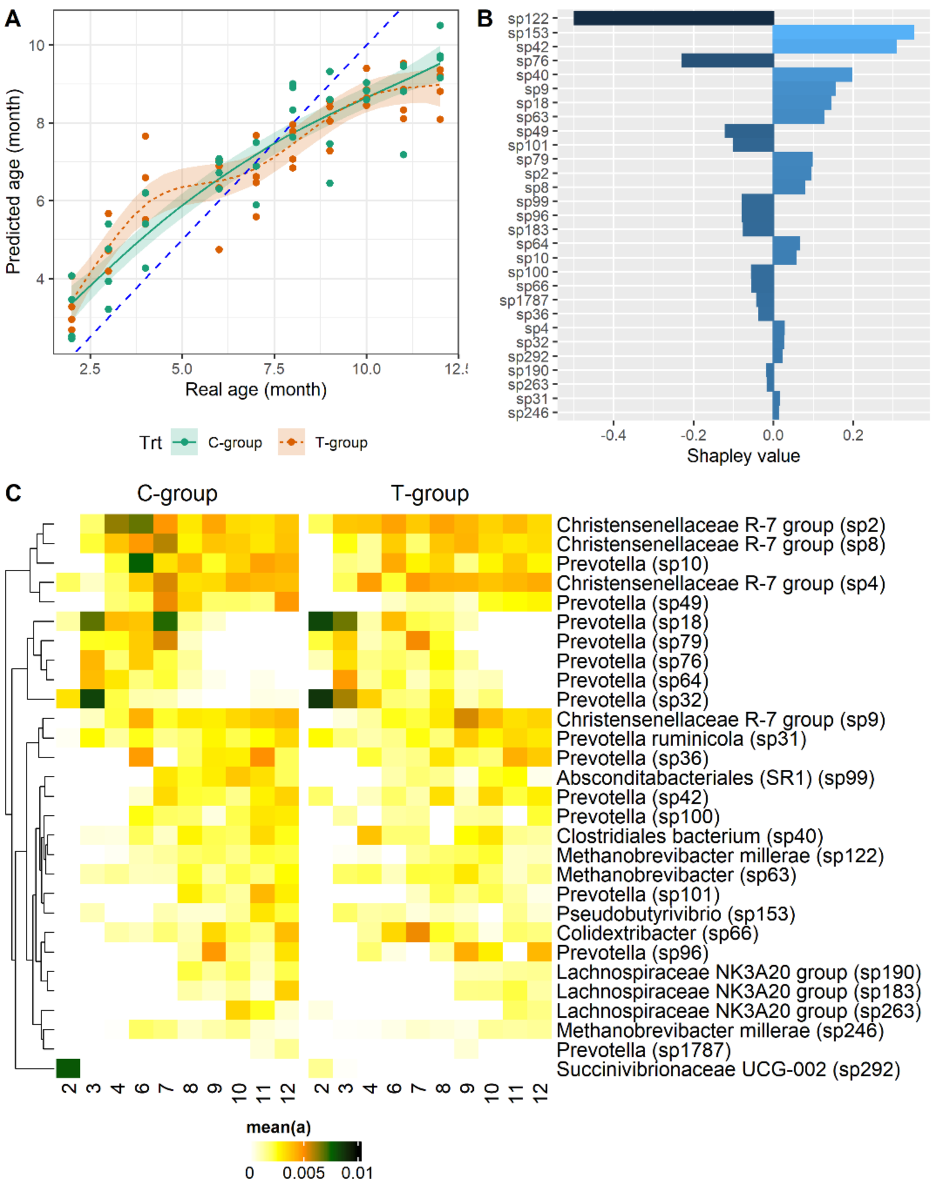

Figure S2. (A) The predicted age of rumen bacteria and archaea communities in treatment (T-group) and control group (C-group) heifers at 2-12 months of age; **(B)** the contribution of species level taxa to the prediction model in month 4, where negative Shapley values reduce the age prediction while positive values increase the prediction. Darker color indicating a smaller and lighter color lager Shapley value; (**C**) the relative abundances of ASVs contributing to predicted age.

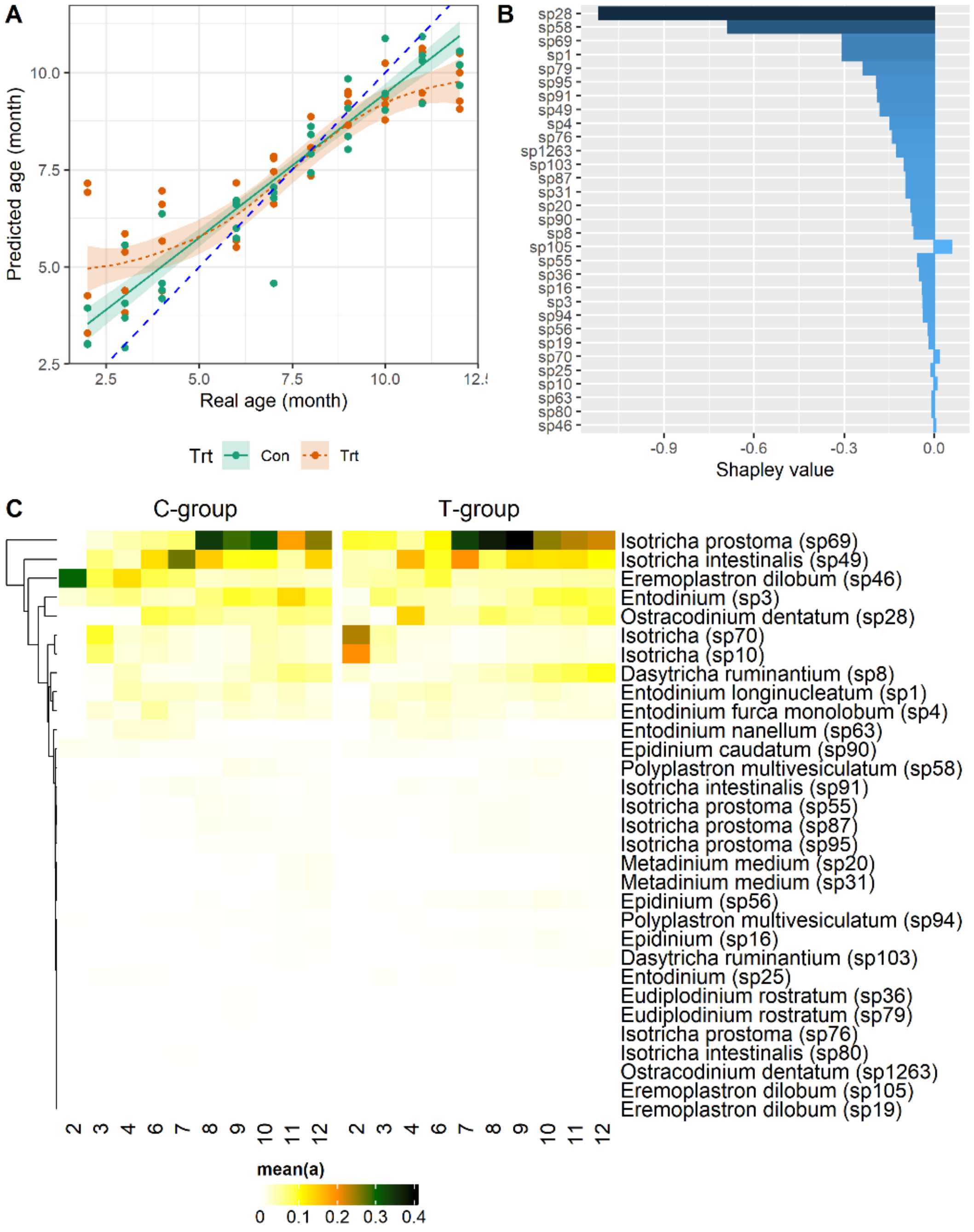

Figure S3. (A) The predicted age of rumen ciliate protozoa communities in treatment (T-group) and control group (C-group) heifers at 2-12 months of age; **(B)** the contribution of species level taxa to the prediction model in month 2, where negative Shapley values reduce the age prediction while positive values increase the prediction. Darker color indicating a smaller and lighter color lager Shapley value; (**C**) the relative abundances of OTUs contributing to predicted age.

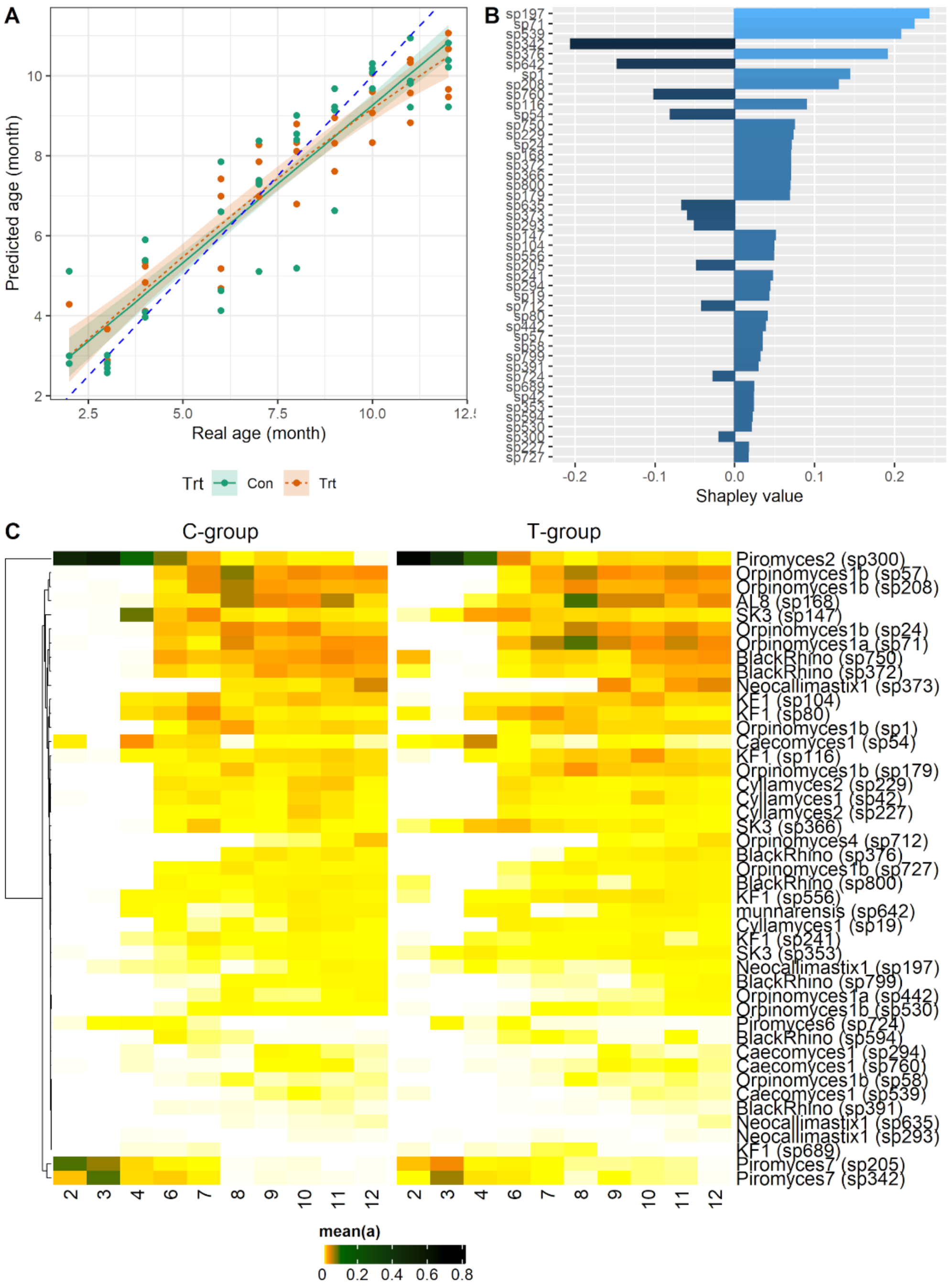

Figure S4. (A) The predicted age of rumen anaerobic fungi community in treatment (T-group) and control group (C-group) heifers at 2-12 months of age; **(B)** the contribution of species level taxa to the prediction model of T-group, where negative Shapley values reduce the age prediction while positive values increase the prediction. Darker color indicating a smaller and lighter color lager Shapley value; (**C**) the relative abundances of OTUs contributing to predicted age.

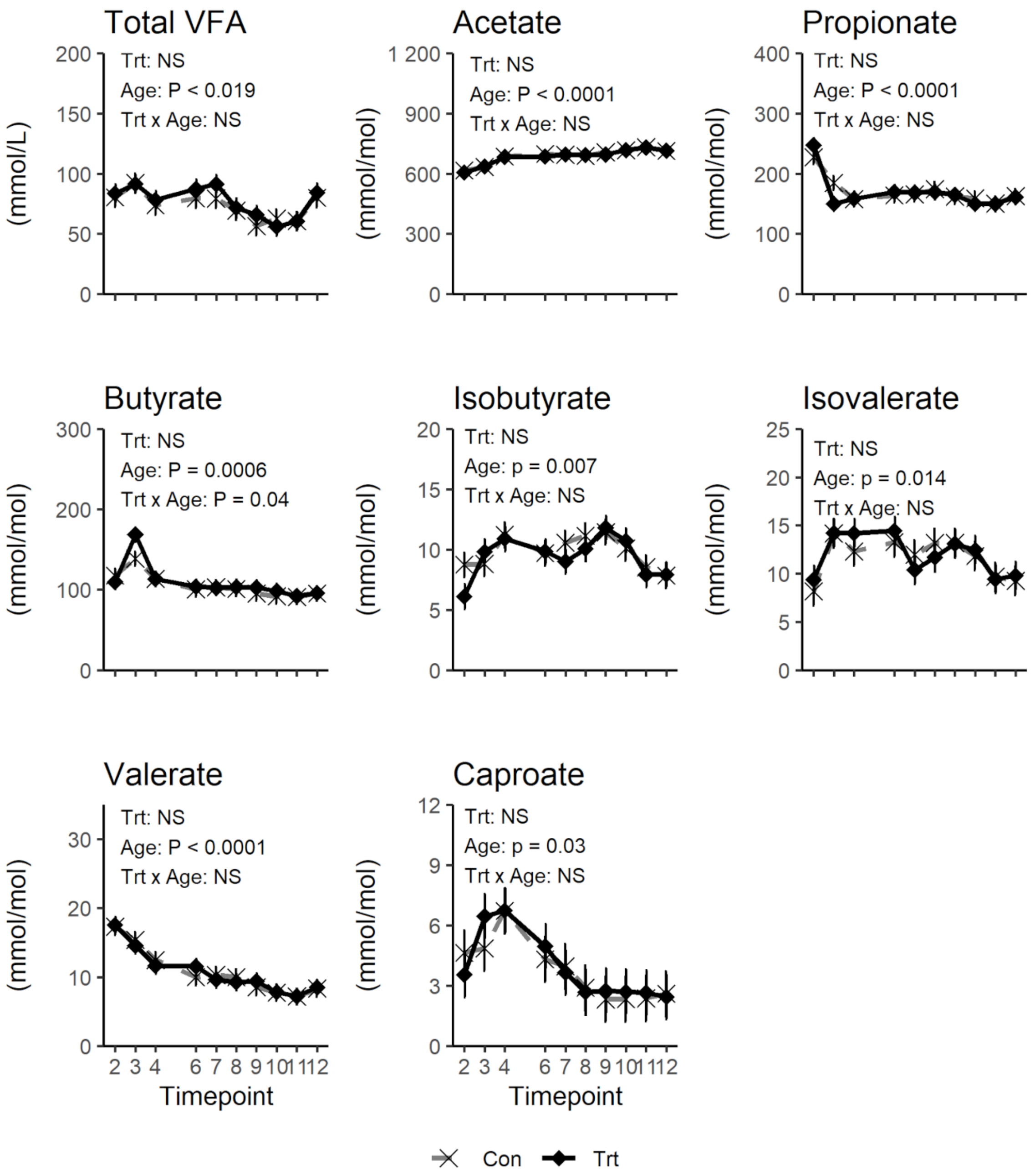

Figure S5. The total volatile fatty acid (VFA) concentration (least square means ± SE) and individual VFA proportions in treatment (Trt) and control group (Con) heifers from weaning (2 months) until 12 months of age. The statistically significant effects of treatment (Trt), age and interaction of age and treatment (Trt x Age) are indicated in annotation.

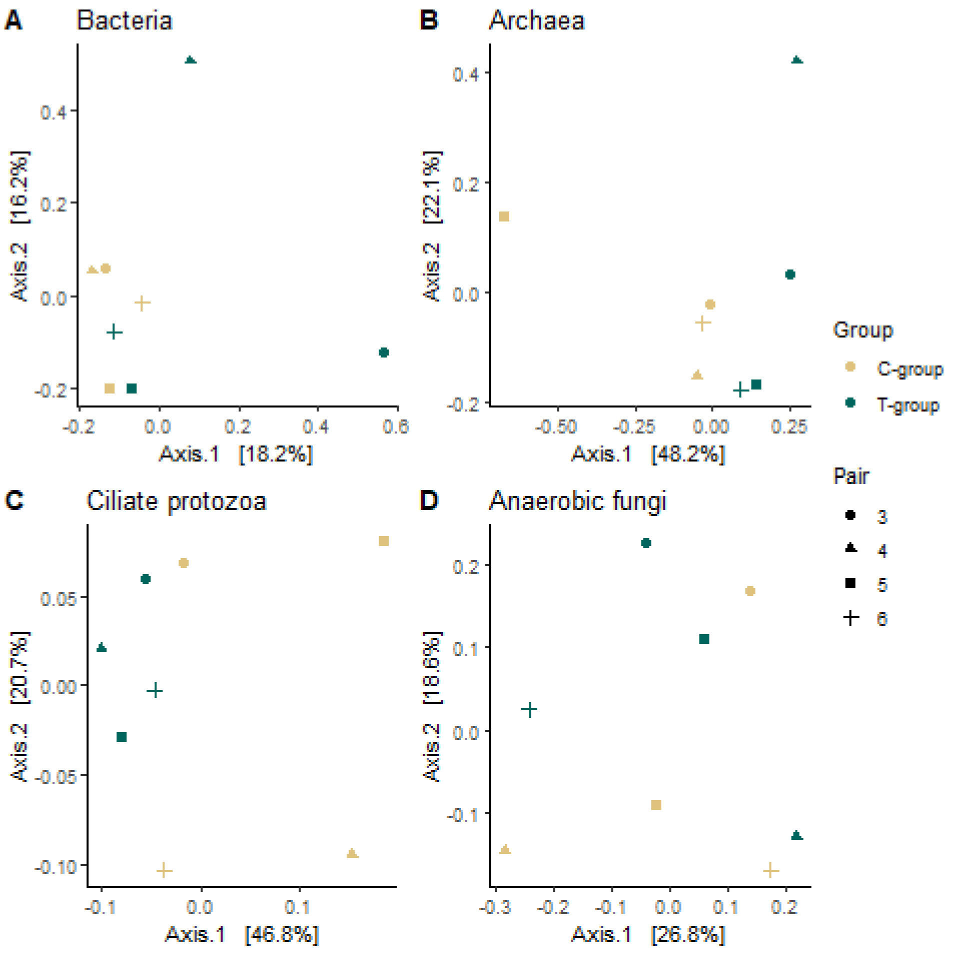

Figure S6. The PCoA visualization of Bray Curtis distances of bacteria (A), archaea (B), protozoa (C), and anaerobic fungi (D) rumen communities in treatment (T-group) and control group (C-group) cows during mid-lactation. Twin-pairs are indicated with shape.

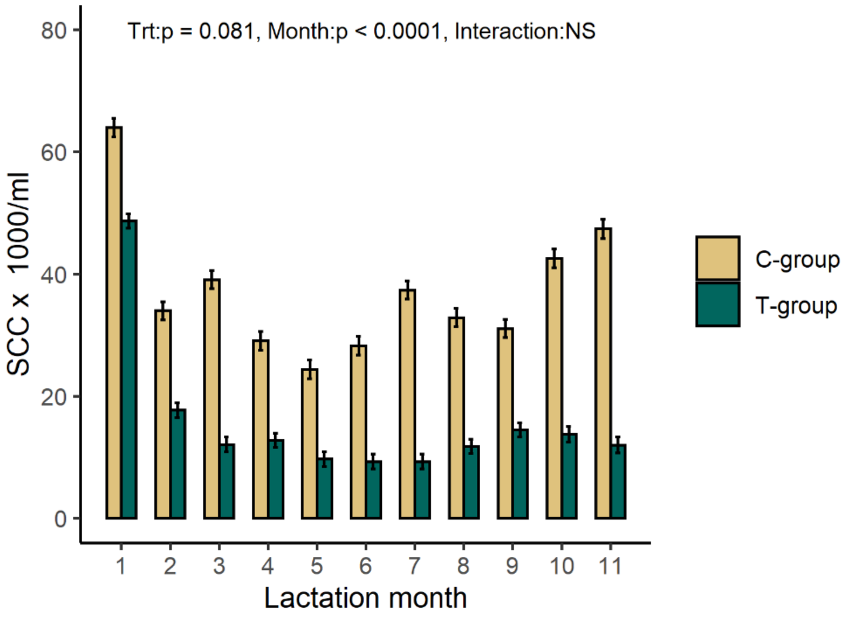

Figure S7. The milk somatic cell count in treatment (T-group) and control group (C-group) cows during the 1st lactation period. Data is presented in least square means ± standard error.

Table S7. The average weight of treatment (T-group) and control group (C-group) heifers at 2-12 months of age. Data is presented in least square means with standard error (SE).

	Weight (kg)		
**Month**	**C-group**	**T-group**	**SE**
2	72.5	77.8	6.7
4	127.2	129.7	9.3
6	158.4	165.3	7.6
7	196.2	194.9	9.2
8	209.0	210.7	7.6
9	238.8	243.3	6.7
10	268.3	272.0	6.7
11	325.8	320.5	6.7
12	346.5	344.5	6.7
**P-value**			
Treatment			0.627
Age			<.0001
Treatment ×Age			0.992

Table S8. The alpha diversity and 16S (bacteria and archaea), 18S (Protozoa) and ITS1 (anaerobic fungi) gene copy numbers in treatment (T-group) and control group (C-group) cows during mid-lactation.

		C-group		T-group		
		Mean	STD	Mean	STD	P-value
Observed ASVs/OTUs						1.00
	Bacteria	664.00	25.47	651.25	216.47	
	Archaea	12.50	5.07	14.75	5.74	0.56
	Protozoa	92.25	6.29	96.75	10.47	0.66
	Fungi	887.25	88.48	927.75	95.29	0.49
Shannon	Bacteria	6.16	0.05	6.05	0.40	0.89
	Archaea	2.19	0.50	2.36	0.39	0.69
	Protozoa	2.96	0.17	3.08	0.05	0.63
	Fungi	3.65	0.26	3.83	0.23	0.69
Simpson	Bacteria	1.00	0.00	1.00	0.00	0.69
	Archaea	0.86	0.07	0.88	0.04	0.69
	Protozoa	0.93	0.02	0.94	0.00	0.34
	Fungi	0.93	0.04	0.95	0.02	0.49
Gene copies per ng/ DNA	Bacteria	3308367	1581920	2488070	269026	0.69
	Archaea	9927	4486	11908	2735	0.89
	Protozoa	40431	6067	44545	11201	0.69
	Fungi	1279	1524	1092	919	1.00

Table S13. The rumen total volatile fatty acid concentration (mol/l) and molar proportions of volatile fatty acids (Mean ± standard deviation (SD)) in treatment (T-group) and control group (C-group) cows during mid-lactation.

	C-group		T-group		
Volatile fatty acids	Mean	SD	Mean	SD	P-values
Total VFA (mol/l)	105.6	14.4	112.7	15.8	0.890
Acetate (mmol/mol)	656.1	29.2	667.6	26.0	0.690
Propionate (mmol/mol)	181.7	13.7	182.	20.6	0.890
Butyrate (mmol/mol)	127.2	19.2	115.6	7.4	0.490
Valerate (mmol/mol)	13.6	1.3	13.1	2.2	0.890
Isovalerate (mmol/mol)	9.4	1.8	10.1	2.1	0.690
Isobutyrate (mmol/mol)	7.7	0.6	6.9	1.3	0.690
Caproate (mmol/mol)	4.4	0.3	5.0	0.6	0.200

Table S14. The body weight (kg), monthly methane production (CH4g), methane yield (CH4 yield) and methane production intensity (CH4 Intensity) in treatment (T-group) and control group (C-group) cows during the first lactation period. Data is presented in least square means ± standard error.

	Body weight (kg)		CH4g		CH4 yield (gCH4/ kg DMI)		CH4 Intensity (gCH4/kg milk)	
Lactation month	C-group	T-group	C-group	T-group	C-group	T-group	C-group	T-group
1	563 ± 26	523 ± 26	281.9 ± 23.6	291.1 ± 28.8	19.1 ± 1.3	16.9 ± 1.6	12.8 ± 1.0	13.5 ± 1.2
2	563 ± 26	532 ± 26	355.5 ± 20.6	334.0 ± 23.6	18.8 ± 1.1	18.2 ± 1.3	14.2 ± 0.8	14.2 ± 1.0
3	563 ± 26	544 ± 26	344.4 ± 20.6	299.6 ± 23.6	17.2 ± 1.1	15.3 ± 1.3	13.6 ± 0.8	12.8 ± 1.0
4	564 ± 26	556 ± 26	336.4 ± 20.6	369.4 ± 23.7	16.6 ± 1.1	18.5 ± 1.3	13.1 ± 0.8	16.1 ± 1.0
5	567 ± 26	568 ± 26	358.8 ± 20.6	387.9 ± 40.1	18.9 ± 1.1	18.0 ± 2.3	14.6 ± 0.8	18.3 ± 1.7
6	572 ± 26	580 ± 26						
7	579 ± 26	592 ± 26						
8	587 ± 26	603 ± 26						
9	597 ± 26	614 ± 26						
10	609 ± 26	624 ± 26						
11	625 ± 26	631 ± 26						
P-values								
Treatment	0.961		0.951		0.498		0.123	
Month	<.0001		0.020		0.358		0.089	
Treatment × month	0.187		0.265		0.378		0.203	

Table S15. The monthly milk lactose, protein and fat percent in treatment (T-group) and control group (C-group) cows during the first lactation period. Data is presented in least square means ± standard error.

Lactation month	Lactose %		Lactose kg		Protein %		Protein kg		Fat %		Fat kg	
	C-group	T-group	C-group	T-group	C-group	T-group	C-group	T-group	C-group	T-group	C-group	T-group
1	4.57 ± 0.05	4.56 ± 0.05	0.98 ± 0.06	0.79 ± 0.06	3.98 ± 0.12	3.98 ± 0.12	0.84 ± 0.05	0.68 ± 0.05	4.70 ± 0.25	4.71 ± 0.25	1.00 ± 0.09	0.81 ± 0.09
2	4.71 ± 0.05	4.72 ± 0.05	1.18 ± 0.06	1.07 ± 0.06	3.71 ± 0.12	3.65 ± 0.12	0.93 ± 0.05	0.83 ± 0.05	4.53 ± 0.25	4.51 ± 0.25	1.14 ± 0.09	1.02 ± 0.09
3	4.68 ± 0.05	4.70 ± 0.05	1.19 ± 0.06	1.10 ± 0.06	3.89 ± 0.12	3.75 ± 0.12	0.99 ± 0.05	0.88 ± 0.05	4.65 ± 0.25	4.45 ± 0.25	1.18 ± 0.09	1.04 ± 0.09
4	4.67 ± 0.05	4.66 ± 0.05	1.20 ± 0.06	1.08 ± 0.06	3.99 ± 0.12	3.86 ± 0.12	1.03 ± 0.05	0.89 ± 0.05	4.57 ± 0.25	4.37 ± 0.25	1.18 ± 0.09	1.01 ± 0.09
5	4.62 ± 0.05	4.61 ± 0.05	1.14 ± 0.06	1.05 ± 0.06	4.01 ± 0.12	3.97 ± 0.12	0.99 ± 0.05	0.90 ± 0.05	4.50 ± 0.25	4.41 ± 0.25	1.11 ± 0.09	1.00 ± 0.09
6	4.56 ± 0.05	4.62 ± 0.05	0.98 ± 0.06	1.01 ± 0.06	3.93 ± 0.12	4.09 ± 0.12	0.84 ± 0.05	0.89 ± 0.05	4.51 ± 0.25	4.54 ± 0.25	0.97 ± 0.09	0.99 ± 0.09
7	4.55 ± 0.05	4.59 ± 0.05	0.91 ± 0.06	0.97 ± 0.06	4.00 ± 0.12	4.13 ± 0.12	0.80 ± 0.05	0.87 ± 0.05	4.46 ± 0.25	4.62 ± 0.25	0.89 ± 0.09	0.98 ± 0.09
8	4.53 ± 0.05	4.58 ± 0.05	0.93 ± 0.06	0.94 ± 0.06	4.07 ± 0.12	4.16 ± 0.12	0.83 ± 0.05	0.85 ± 0.05	4.50 ± 0.25	4.66 ± 0.25	0.93 ± 0.09	0.95 ± 0.09
9	4.53 ± 0.05	4.58 ± 0.05	0.92 ± 0.06	0.89 ± 0.06	4.10 ± 0.12	4.19 ± 0.12	0.83 ± 0.05	0.81 ± 0.05	4.70 ± 0.25	4.70 ± 0.25	0.96 ± 0.09	0.91 ± 0.09
10	4.54 ± 0.05	4.56 ± 0.06	0.90 ± 0.06	0.84 ± 0.06	4.19 ± 0.12	4.19 ± 0.12	0.83 ± 0.05	0.77 ± 0.06	5.07 ± 0.25	4.75 ± 0.26	1.01 ± 0.09	0.87 ± 0.09
11	4.54 ± 0.06	4.54 ± 0.06	0.80 ± 0.07	0.86 ± 0.07	4.30 ± 0.13	4.19 ± 0.13	0.76 ± 0.06	0.79 ± 0.06	5.27 ± 0.27	4.76 ± 0.27	0.92 ± 0.09	0.90 ± 0.09
P-values												
Treatment	0.176		0.595		0.996		0.498		0.678		0.494	
Month	<.0001		<.0001		<.0001		<.0001		0.013		<.0001	
Treatment x Month	0.737		0.002		0.232		0.001		0.281		0.001	
